# Melatonin suppresses ER stress-dependent proapoptotic effects via AMPK in bone mesenchymal stem cells during mitochondrial oxidative damage

**DOI:** 10.1186/s13287-020-01948-5

**Published:** 2020-10-15

**Authors:** Chongxi Fan, Jianyu Feng, Chi Tang, Zhengbin Zhang, Yingtong Feng, Weixun Duan, Mingming Zhai, Zedong Yan, Liwen Zhu, Lele Feng, Hanzhao Zhu, Erping Luo

**Affiliations:** 1 Department of Military Biomedical Engineering, Air Force Medical University, 169 Changle West Road, Xi’an, 710032 China; 2 Department of Oncology, Air Force Medical Center of PLA, 30 Fucheng Road, Beijing, 100142 China; 3 Department of Cardiovascular Surgery, Xijing Hospital, Air Force Medical University, 127 Changle West Road, Xi’an, 710032 China; 4 Department of Geriatrics, The 8th Medical Center of Chinese PLA General Hospital, 17 Heishanhu Street, Beijing, 100091 China; 5 Department of Thoracic Surgery, Tangdu Hospital, Air Force Medical University, 1 Xinsi Road, Xi’an, 710038 China; 6 Department of Cardiology, The First Affiliated Hospital of Xi’an Medical University, 277 Yanta West Road, Xi’an, 710077 China

**Keywords:** Melatonin, Bone marrow mesenchymal stem cells, ER stress, AMPK, Oxidative stress

## Abstract

**Background:**

Bone marrow mesenchymal stem cells (BMSCs) have been used as important cell-based tools for clinical applications. Oxidative stress-induced apoptosis causes a low survival rate after transplantation, and the underlying mechanisms remain unknown. The endoplasmic reticulum (ER) and mitochondria are vital organelles regulated by adenosine monophosphate (AMP)-activated protein kinase (AMPK), especially during oxidative stress injury. Melatonin exerts an antioxidant effect by scavenging free radicals. Here, we aimed to explore whether cytoprotective melatonin relieves ER stress-mediated mitochondrial dysfunction through AMPK in BMSCs after oxidative stress injury.

**Methods:**

Mouse BMSCs were isolated and exposed to H_2_O_2_ in the absence or presence of melatonin. Thereafter, cell damage, oxidative stress levels, mitochondrial function, AMPK activity, ER stress-related proteins, and apoptotic markers were measured. Additionally, the involvement of AMPK and ER stress in the melatonin-mediated protection of BMSCs against H_2_O_2_-induced injury was investigated using pharmacologic agonists and inhibitors.

**Results:**

Melatonin improved cell survival and restored mitochondrial function. Moreover, melatonin intimately regulated the phosphorylation of AMPK and molecules associated with ER stress pathways. AMPK activation and ER stress inhibition following melatonin administration improved the mitochondrial membrane potential (MMP), reduced mitochondria-initiated oxidative damage, and ultimately suppressed apoptotic signaling pathways in BMSCs. Cotreatment with *N*-acetyl-l-cysteine (NAC) significantly enhanced the antioxidant effect of melatonin. Importantly, pharmacological AMPK activation/ER stress inhibition promoted melatonin-induced cytoprotection, while pharmacological AMPK inactivation/ER stress induction conferred resistance to the effect of melatonin against H_2_O_2_ insult.

**Conclusions:**

Our data also reveal a new, potentially therapeutic mechanism by which melatonin protects BMSCs from oxidative stress-mediated mitochondrial apoptosis, possibly by regulating the AMPK-ER stress pathway.

## Background

Bone marrow mesenchymal stem cells (BMSCs) are multipotent stromal cells that have been used as important cell-based tools for various clinical applications due to their high proliferative capacity and their potential to differentiate into multiple lineages, including the bone, cartilage, and fat [1]. Unfortunately, the survival rate of BMSCs in the circulation or injury microenvironment after transplantation is low as a result of hydrodynamic shear stress, excessive inflammatory cytokines, and oxidative stress [2–4].

Although these primary causes of cell death occur together in BMSCs, endoplasmic reticulum (ER) stress has emerged as a potential effector of these events [5, 6]. The ER, which functions as a site for lipid biosynthesis, protein folding and assembly, and cellular divalent calcium cation (Ca ^2+^) storage in eukaryotic cells, is also a major signal-transducing organelle that senses cellular stress under homeostasis [7]. Under stress conditions, the unfolded protein response (UPR) serves as an adaptive response for degrading misfolded proteins that accumulate in the ER, whereas uncontrolled or prolonged ER stress can trigger cell apoptosis; the coupling of these responses in specialized cells or tissues is now thought to be fundamental in oxidative stress injury [8, 9]. Furthermore, ER stress is initiated by three ER transmembrane proteins: RNA-dependent protein kinase (PKR)-like ER kinase (PERK), inositol-requiring protein 1 (IRE1), and activating transcription factor 6 (ATF6) [10].

Mitochondria are organelles that respond to cell apoptosis regulatory signals by selectively releasing cytochrome C (Cyto-C) or by increasing reactive oxygen species (ROS) production and decreasing the mitochondrial membrane potential (MMP), which may further worsen ER dysfunction and enhance cell death [11]. However, the upstream mediator of mitochondrial and ER damage has not been identified. Mitochondria are also considered to be producers of energy through adenosine triphosphate (ATP) synthesis, which plays a critical role in the regulation of energy metabolism through adenosine monophosphate (AMP)-activated protein kinase (AMPK) signaling [12, 13].

AMPK is an evolutionarily conserved energy sensor associated with an elegant energy metabolism system; it has diverse cytoprotective effects on glucose and lipid metabolism, the cell cycle, and protein synthesis and participates in cell apoptosis [14, 15]. It is believed that increased AMP levels, ATP deprivation, and oxidative stress can activate AMPK [16]. A growing body of evidence suggests that AMPK specifically regulates various aspects of mitochondrial biology and homeostasis, including mitochondrial numbers, through stimulation of mitochondrial biogenesis and control of the shape of the mitochondrial network and the quality of mitochondria in cells [17]. In addition, although Lu and colleagues showed that elevated circulating saturated fatty acid concentrations induce ER stress-mediated apoptosis, which is blocked by AMPK activation [18], in human BMSCs, whether AMPK is a pivotal modulator of ER stress during mitochondrial oxidative damage remains unaddressed. In addition, identifying an effective and rapid therapeutic approach to attenuate the influence of the circulatory or injury microenvironment on BMSCs, especially one that involves endogenous active ingredients, remains an urgent clinical need.

Melatonin (*N*-acetyl-5-methoxytryptamine, Mel) is an indoleamine-containing hormone that is secreted mainly from the pineal gland in mammals, and its existence in other tissues, including the ovary, testis, gut, placenta liver, and especially the bone marrow, where it is found at high levels, has also been verified [19]. Many studies have demonstrated that melatonin regulates a variety of important physiological functions, such as circadian rhythms and reproductive and neuroendocrine activities [20]. In recent years, increasing evidence has focused on the antioxidant and anti-inflammatory effects of melatonin on BMSCs after injury. For example, Lee et al. demonstrated that treatment with melatonin suppresses the activation of ER stress-associated proteins and the occurrence of apoptotic cell death in human adipose-derived mesenchymal stem cells (MSCs), which enhances the therapeutic efficacy of MSCs in a murine hindlimb ischemia model [21]. Furthermore, melatonin maintains human MSC survival and promotes osteogenic differentiation in the IL-1β-induced inflammatory environment, suggesting that melatonin treatment could be a promising method for bone regenerative engineering [22]. Importantly, our findings indicate that melatonin contributes to the amelioration of high-flow shear stress-induced BMSC injury by activating melatonin receptors and AMPK/acetyl-Co A carboxylase (ACC) signaling [4]. Therefore, this study, as an extension of our previous research, focuses on the ability of cytoprotective melatonin to relieve ER stress-mediated mitochondrial dysfunction through AMPK in mouse BMSCs after hydrogen peroxide (H_2_O_2_)-induced oxidative stress injury.

## Methods

### Reagents and antibodies

Melatonin (> 98% purity, M5250), 4-phenylbutyric acid (4-PBA, > 99% purity, SML0309), and dimethyl sulfoxide (DMSO, D8418) were purchased from Sigma-Aldrich (St. Louis, MO, USA); thapsigargin (TG, > 98% purity, ab120286) was from Abcam (Cambridge, MA, USA); compound C dihydrochloride (CpC, T6146) and acadesine (AICAR, T1477) were from TargetMol (Wellesley Hills, MA, USA); *N*-acetyl-l-cysteine (NAC, S1623) was from Selleck Chemicals (Houston, TX, USA); and 5,5′,6,6′-tetraethyl-benzimidazolylcarbocyanine iodide (JC-1, C2006), the bicinchoninic acid (BCA, P0011) protein assay kit, the crystal violet staining solution (C0121), the BeyoClick™ EdU cell proliferation kit with Alexa Fluor 594 (C0078S), 4′,6-diamidino-2-phenylindole (DAPI, C1002), the commercial assay kit for lactate dehydrogenase (LDH, C0016) analysis, the cell mitochondria isolation kit (C3601), immune staining wash buffer (Triton X-100, P0106), radioimmunoprecipitation assay (RIPA) lysis buffer (P0013C), and SDS-PAGE sample loading buffer (5×, P0015) were from Beyotime Biotechnology (Shanghai, China). MitoSOX™ Red mitochondrial superoxide indicator (M36008) was from Molecular Probes Inc. (Invitrogen, Eugene, OR, USA). The small interfering RNA (siRNA) oligonucleotides against AMPK (sc-29674) and DDIT3 (sc-35438), the nonspecific control siRNA oligonucleotide (sc-37007), and the transfection reagent (sc-29528) were purchased from Santa Cruz Biotechnology (Dallas, Texas, 75220, USA), and 30% H_2_O_2_ was purchased from Tianjin Beilian Fine Chemical Development Co., Ltd. (Tianjin, China). C57BL/6 mouse MSC basal medium (BM, MUBMX-90011), osteogenic differentiation basal medium (MUBMX-90021), and adipogenic differentiation basal medium (MUBMX-90031) were from Cyagen Biosciences (Soochow, China); penicillin-streptomycin solution (15140122) was purchased from Life Technologies Co., Ltd. (Carlsbad, CA, USA), and TRIzol reagent was from Invitrogen (Carlsbad, CA, USA). HiScript II Q RT SuperMix (R223-01) and ChamQ SYBR qPCR Master Mix (Q311-02) were purchased from Vazyme Biotech Co., Ltd. (Nanjing, China), and 0.25% trypsin-ethylenediaminetetraacetic acid (EDTA) solution (T1300), Hanks balanced salt solution (HBSS) with calcium and magnesium (H1025), and bovine serum albumin (BSA, A8010) were purchased from Solarbio Science & Technology Co., Ltd. (Beijing, China). The Cell Counting Kit-8 (CCK-8, C008-3) assay kit was obtained from 7Sea Pharmatech Co., Ltd. (Shanghai, China). The terminal deoxynucleotidyl transferase dUTP nick end labeling (TUNEL, 11684795910) staining kit for apoptosis detection was from Roche Molecular Biochemicals (Mannheim, Germany), and the Annexin V-FITC/propidium iodide (PI) staining kit (40302ES50) and reactive oxygen species (50101ES01) assay kit were acquired from Yeasen Biotech Co., Ltd. (Shanghai, China). The flow cytometry staining buffer (00-4222-26) was from eBioscience (San Diego, CA, USA). The antibodies and primers used in this study are listed in Tables 1 and 2. Other chemicals and reagents were of analytical grade.

**Table 1:** List of antibodies

Target proteins	Cat. no.	Source	Mol.Wt.	Provider	Application
CD16/32	14-0161	Rat	N/A	eBioscience (San Diego, CA, USA)	FACS
Phycoerythrin (PE) anti-mouse/rat CD29 Antibody	102207	Armenian Hamster	N/A	BioLegend (San Diego, CA, USA)	FACS
PE anti-mouse CD34 Antibody	128609	Armenian Hamster	N/A	BioLegend (San Diego, CA, USA)	FACS
PE anti-mouse/human CD44 Antibody	103007	Rabbit	N/A	BioLegend (San Diego, CA, USA)	FACS
PE anti-mouse CD45 Antibody	103105	Rabbit	N/A	BioLegend (San Diego, CA, USA)	FACS
PE anti-mouse CD73 Antibody	127205	Rabbit	N/A	BioLegend (San Diego, CA, USA)	FACS
PE anti-mouse CD105 Antibody	120407	Rabbit	N/A	BioLegend (San Diego, CA, USA)	FACS
PE Armenian Hamster IgG Isotype Ctrl Antibody	400907	Armenian Hamster	N/A	BioLegend (San Diego, CA, USA)	FACS
PE Rat IgG2a, κ Isotype Ctrl Antibody	400507	Rabbit	N/A	BioLegend (San Diego, CA, USA)	FACS
PE Rat IgG2b, κ Isotype Ctrl Antibody	400607	Rabbit	N/A	BioLegend (San Diego, CA, USA)	FACS
APAF-1	21710-1-AP	Rabbit	142 kDa	ProteinTech (Wuhan, China)	WB
phospho-PERK (p-PERK)	DF7576	Rabbit	125 kDa	Affinity Biosciences (Cincinnati, OH, USA)	WB
PERK	20582-1-AP	Rabbit	125 kDa	ProteinTech (Wuhan, China)	WB
BiP	ab21685	Rabbit	78 kDa	Abcam (Cambridge, MA, USA)	WB & IF
Calreticulin (CRT)	ab22683	Mouse	N/A	Abcam (Cambridge, MA, USA)	IF
phospho-AMPK (p-AMPK)	#2535	Rabbit	62 kDa	Cell Signaling Technology (Beverly, MA, USA)	WB
AMPK	#2532	Rabbit	62 kDa	Cell Signaling Technology (Beverly, MA, USA)	WB
phospho-eIF2α (p-eIF2α)	#3398	Rabbit	38 kDa	Cell Signaling Technology (Beverly, MA, USA)	WB
eIF2α	#5324	Rabbit	38 kDa	Cell Signaling Technology (Beverly, MA, USA)	WB
Caspase-3 (Casp-3)	#9665	Rabbit	35, 19, 17 kDa	Cell Signaling Technology (Beverly, MA, USA)	WB
Cleaved-Caspase-3 (C-Casp-3)	#9664	Rabbit	19, 17 kDa	Cell Signaling Technology (Beverly, MA, USA)	WB & IF
DNA damage-inducible transcript 3 (DDIT3)	ab11419	Mouse	31 kDa	Abcam (Cambridge, MA, USA)	WB
Bcl2	12789-1-AP	Rabbit	26 kDa	ProteinTech (Wuhan, China)	WB
MnSOD	ab13533	Rabbit	25 kDa	Abcam (Cambridge, MA, USA)	WB
Bax	#2772	Rabbit	20 kDa	Cell Signaling Technology (Beverly, MA, USA)	WB
Cytochrome C oxidase IV (COX IV)	11242-1-AP	Rabbit	18–17 kDa	ProteinTech (Wuhan, China)	WB
Cytochrome C (Cyto-C)	ab13575	Mouse	12 kDa	Abcam (Cambridge, MA, USA)	WB
β-Tubulin	10094-1-AP	Rabbit	55 kDa	ProteinTech (Wuhan, China)	WB
β-actin	SC-47778	Mouse	43 kDa	Santa Cruz (Dallas, TX, USA)	WB
GAPDH	60004-1-Ig	Mouse	36 kDa	ProteinTech (Wuhan, China)	WB
HRP-conjugated goat anti-mouse antibody	ZB-2305	Goat	N/A	Zhongshan Company (Beijing, China)	WB
HRP-conjugated goat anti-rabbit antibody	ZB-2301	Goat	N/A	Zhongshan Company (Beijing, China)	WB
Alexa Fluor 488-conjugated goat anti-mouse IgG	EK011	Goat	N/A	Zhuangzhibio (Xi’an, China)	IF
Cy3-conjugated goat anti-rabbit IgG	EK022	Goat	N/A	Zhuangzhibio (Xi’an, China)	IF

**Table 2:** List of primers

Target genes	Forward sequence	Reverse sequence	Provider
*Apaf-1*	AGTAATGGGTCCTAAGCATGTTG	GCGATTGGGAAAATCACGTAAAA	Rescript (Nanjing, China)
*GAPDH*	AGGTCGGTGTGAACGGATTTG	TGTAGACCATGTAGTTGAGGTCA	Rescript (Nanjing, China)

### Ethics statement

All animal experimental procedures performed in the present study were conducted in accordance with the Guide for the Care and Use of Laboratory Animals published by the US National Institutes of Health (National Institutes of Health Publication No. 85-23, revised in 1996), and approval was obtained from the Ethics Committee of the Fourth Military Medical University (No. 20180301).

### Animals

Adult male C57BL/6 mice (8-week old, 20–25 g) were obtained from the animal center of Air Force Medical University (Xi’an, China). The mice were kept in an Association for Assessment and Accreditation of Laboratory Animal Care (AAALAC)-accredited facility in an environmentally controlled room (12-h light-dark cycle, 60% humidity) at 20-23 °C, and they were allowed ad libitum access to standard chow and sterile water.

### Isolation and culture of mouse BMSCs

The mouse BMSCs were flushed from the tibial and femoral marrow compartments under sterile conditions with 3% fetal bovine serum (FBS, Cyagen Biosciences, Soochow, China) in PBS, and the procedure adhered strictly to standard protocols [23]. After passage through a 70-μm cell strainer (Corning, NY, USA), all the cells were cultured with C57BL/6 mouse BM with 10% FBS in a standard CO_2_ incubator (95% air, 5% CO_2_). MSCs were isolated through frequent and gentle medium changes within 2 weeks, and adherent marrow cells were subcultured to passage 4 (P4). Cells at less than passage 10 were used for subsequent trials, according to previous recommendations [23].

### Flow cytometric BMSC characterization

To identify the cell surface markers of BMSCs, P4 cells were collected according to the previous literature, detached from plates with 0.25% trypsin-EDTA solution, and gently resuspended in flow cytometry staining solution at a density of 5 × 10^6^ cells/mL [3, 24]. Then, the following antibodies were applied for 30 min at room temperature protected from light: anti-CD29-PE, anti-CD34-PE, anti-CD44-PE, anti-CD45-PE, anti-CD73-PE, and anti-CD105-PE. The nonspecific Fc receptor was blocked with anti-CD16/32 antibodies. Nonspecific fluorescence was determined by incubation with isotype-matched anti-mouse monoclonal antibodies. After staining, the samples were processed with EPICS XL-MCL (Beckman Coulter, Brea, CA, USA), and the analysis was performed with EXPO32 ADC.

### Adipogenesis and osteogenesis in mouse BMSCs

Adipogenic and osteogenic differentiation of BMSCs was performed strictly according to the commercial product manual from Cyagen Biosciences (Soochow, China). Briefly, BMSCs (P6) were seeded in six-well plates (precoated with 5% gelatin) at a density of 2 × 10^4^ cells/cm^2^ at 37 °C with 5% CO_2_ for at least 12 h. For osteogenic differentiation, 2 mL of preheated complete inducing medium was replaced every 3 days when the percentage of cell fusion reached 60–70%. After 2–4 weeks of induction, depending on the morphological changes and growth of the cells, the cells were stained with Alizarin Red. For adipogenesis, BMSCs were incubated with adipogenic differentiation medium A until they reached 100% confluence or fusion. After 2–3 days, the supernatant was replaced with adipogenic differentiation medium B for 1 day. During 3–5 alternating cycles (12–20 days), medium B was used for 4–7 days until the lipid droplets became sufficiently large and round. Adipogenesis was determined by staining with Oil Red O solution to visualize lipids in the cytoplasm.

### Experimental conditions

Step one: The cytotoxic effects of H_2_O_2_-induced oxidative stress on mouse BMSCs and the regulation of AMPK and ER stress during this process were first evaluated. The cells were incubated in normal culture medium in 96-well plates until they reached 80% confluence. The medium was then replaced with different concentrations of H_2_O_2_ (0–800 μM, diluted in serum-free BM) for another 24 h to explore suitable conditions for subsequent experimental modeling. Step two: The cytoprotective effects of melatonin were confirmed. The maximum safe dose of melatonin for BMSCs has been studied, and this dose was used in experiments investigating the ability of melatonin to protect against H_2_O_2_-induced injury. Step three: Some small-molecule modulators (such as Met, Cpc, TG, and 4-PBA) and siRNA agents were used at the indicated doses to modulate their target proteins to further investigate the roles of AMPK and ER stress in the protective effect of melatonin against oxidative stress damage in BMSCs. Unless otherwise stated, all the compounds were dissolved in DMSO or PBS. The solution was stored at 4 °C for 7 days, diluted in BM (without FBS), and filtered immediately before the experiment to achieve a DMSO concentration of less than 0.1%.

### Cell viability analysis

Mouse BMSCs in logarithmic phase were inoculated in 96-well plates (precoated with 5% gelatin) at a density of 3 × 10^4^ cells/cm^2^ at 37 °C with 5% CO_2_ for at least 12 h to achieve a stable state. Then, different treatments were implemented at the indicated doses for the indicated times. Ultimately, 100 μL of BM with 10 μL of CCK-8 solution was added and incubated at 37 °C with 5% CO_2_ for 2 h in the dark. The optical density (OD) values were analyzed at 450 nm using a microplate reader (SpectraMax 190, Molecular Device, USA) within 3 min.

### Cell proliferation assay

Cell proliferation was assessed with an EdU-Alexa Fluor 594 staining kit according to the manufacturer’s instructions. Briefly, microscope slides with BMSCs were prepared and incubated with 10 μM EdU for 2 h at 37 °C in the dark. Then, the samples were fixed with 4% paraformaldehyde at room temperature for 15 min, followed by 0.3% Triton X-100 for another 10 min. Finally, click additive solution was used to excite fluorescence (excitation/emission = 590/615 nm), and the nuclei were visualized with Hoechst 33342 (excitation/emission = 346/460 nm). The fluorescence signals in at least five random separate fields were acquired using an FV1000 Olympus confocal microscope (Olympus, Tokyo, Japan). The fluorescence intensity was analyzed with ImageJ software (1.52a, National Institutes of Health, Bethesda, MD, USA).

### LDH cytotoxicity assay

Oxidative stress-induced cell death was assessed spectrophotometrically via an LDH release assay of cell culture supernatants according to the manufacturer’s instructions. The absorbance of all samples was read at 490 nm using a SpectraMax M5 microplate reader (SpectraMax M5, Molecular Device, USA) within 3 min.

### Determination of BMSC apoptosis

The cell apoptosis rate was detected using the TUNEL and Annexin V-FITC/PI staining kits after different treatments. For TUNEL analysis, BMSCs were grown on gelatin-precoated microscopy slides and fixed with 4% paraformaldehyde at room temperature for 15 min. The samples were then covered with 100 μL of mixed TUNEL reagent for 1 h at 37 °C in a thermostatic water bath. DAPI solution (5 μg/mL) was then added to reveal the number of nuclei. The TUNEL signals were observed with an FV1000 Olympus confocal microscope (Olympus, Tokyo, Japan). At least 100 cells/fields (5 fields in general) were randomly counted to determine the ratio of the number of TUNEL-positive nuclei to the number of DAPI-positive nuclei and calculate apoptosis rates.

For Annexin V-FITC/PI staining, BMSCs were seeded in six-well plates at 6 × 10^5^ cells/mL for overnight stabilization. After administration, the cells were gently collected with trypsin solution. The suspension was centrifuged at 300 g and 4 °C for 5 min. In total, 1 × 10^5^ cells were resuspended in 195 μL of FITC-conjugated annexin V binding buffer containing 5 μL of Annexin V-FITC and 10 μL of PI at room temperature for 20 min in the dark and maintained on ice when processing. The samples were analyzed with EPICS XL-MCL (Beckman Coulter, Brea, CA, USA), and analysis was performed with EXPO32 ADC within 30 min.

### Determination of intracellular free radical production

The ROS in BMSCs were measured with DCFH-DA staining on gelatin-precoated microscopy slides without fixation at 37 °C for 30 min in the dark. The fluorescence signals in at least five random separate fields were acquired using an FV1000 Olympus confocal microscope (Olympus, Tokyo, Japan) at a wavelength of 488/525 nm (excitation/emission). The fluorescence intensity was analyzed with ImageJ software (1.52a, National Institutes of Health, Bethesda, MD, USA).

### Mitochondrial superoxide analysis

The MitoSOX™ Red indicator is a novel fluorochrome for the highly selective detection of superoxide in the mitochondria of live cells on glass coverslips after different treatments. The cells were incubated with 5 μM MitoSOX™ reagent working solution (stored in DMSO at 5 mM and diluted with HBSS before use) for 10 min at 37 °C, and the samples were protected from light. The fluorescence signal was imaged with an FV1000 Olympus confocal microscope (Olympus, Tokyo, Japan) at an excitation/emission = 510/580 nm. The fluorescence intensity was analyzed with ImageJ software (1.52a, National Institutes of Health, Bethesda, MD, USA).

### MMP detection

The levels of MMP in BMSCs were analyzed by staining with the cationic dye JC-1 according to our previous study [25]. JC-1 aggregates in the mitochondrial matrix predominantly produce red fluorescence, as assessed using excitation/emission wavelengths of 525/590 nm; otherwise, after mitochondrial polarization, JC-1 is in the monomer form, which can produce green fluorescence at 490/530 nm. The specimens were incubated with JC-1 solution for 20 min at 37 °C in the dark. The MMP was represented by the ratio of red fluorescence intensity to green fluorescence intensity.

### Transwell chemotaxis assays

BMSCs were cultured in Transwell chambers with 8-μm pores (3422; Corning Inc., ME, USA). The upper chamber was seeded with MSCs (2 × 10^4^ cells), which were grown in 100 μL of BM. Additionally, the lower chamber contained 600 μL of BM with various concentrations of melatonin [24]. After 24 h, the cells remaining on the upper surface of the filter were removed with cotton swabs, and those that traversed to the lower surface were revealed by crystal violet staining solution for 30 min at room temperature. The numbers of migrated cells were counted in five randomly selected fields using a 600D camera (Canon Company, Tokyo, Japan).

### Scratch migration assays

BMSCs were plated on six-well plates and grown until they reached close to 90% confluence. After washing with PBS, a scratch was created in the cell layer along the diameter of the well with a 200-μL pipet tip. Next, the cells were incubated with 2 mL of serum-free BM containing various concentrations of melatonin. Three photomicrographs of each scratch were obtained at the initial time (0 h) of wound creation, and the same location was photographed 24 h later to determine the extent of wound closure [26]. ImageJ software (1.52a, National Institutes of Health, Bethesda, MD, USA) was used to measure the relative width of the scratch in each group.

### Immunofluorescence microscopy

After treatment with the indicated agents for the indicated times, the BMSCs on coverslips were washed with PBS and fixed in 4% paraformaldehyde at room temperature for 15 min. Then, 3% BSA in PBS containing 0.3% Triton X-100 solution was used to increase the membrane permeability and prevent nonspecific adsorption of immune agents at 37 °C for 1 h. The primary antibodies were incubated overnight at 4 °C. On the following day, Alexa Fluor 488-conjugated goat anti-mouse IgG antibodies against calreticulin (CRT) and Cy3-conjugated goat anti-rabbit IgG antibodies against Bip were used to label the cells at 37 °C for 1 h. DAPI solution (5 μg/mL) was added to stain the cell nuclei. The fluorescence signal was captured with an FV1000 Olympus confocal microscope (Olympus, Tokyo, Japan) at excitation/emission = 493/519 nm (Alexa Fluor 488) and excitation/emission = 550/570 nm (Cy3). The fluorescence intensity was analyzed with ImageJ software (1.52a, National Institutes of Health, Bethesda, MD, USA). The primary antibodies used in the present study are shown in Table 1.

### Cytochrome C (Cyto-C) release assay

The mitochondrial and cytosolic fractions were separated according to the manufacturer’s instructions. Briefly, BMSCs were plated in six-well plates and treated with the indicated agents. Cells were collected with 0.25% trypsin-EDTA solution, washed with ice-cold PBS, and resuspended in homogenization buffer containing 1 mM PMSF for 15 min. The cells were homogenized approximately 10–30 times using a Teflon/glass tissue grinder. The effect of the homogenate was evaluated with trypan blue. The cell lysate was centrifuged at 600 g for 10 min to remove unbroken cells and nuclei, and the supernatant was centrifuged at 11,000 g for 10 min at 4 °C to obtain the supernatant (without touching the precipitate) and the pure pellet. The supernatant was used as the cytosolic fraction, whereas the pellet containing the mitochondria was washed and resuspended in homogenization buffer. Protein concentrations were estimated with the BCA protein assay kit. The translocation of Cyto-C from mitochondria to the cytoplasm was further detected by western blotting.

### Knockdown of gene expression with siRNA

BMSCs were cultured in six-well plates in the presence of 10% FBS without antibiotics for 6 h. The working solution (containing targeted-siRNA oligonucleotides and transfection reagent) was prepared at room temperature for 30 min. The cells were then cultured in the above solution with culture medium for 48 h to allow maximal inhibition of protein expression. Scrambled small interfering RNA (Consi) was used as the control. The knockdown efficiency was further assessed by western blotting.

### Western blot analysis

The protein lysates from BMSCs were prepared after different treatments. Equal amounts of protein (approximately 20–35 μg) were loaded onto either 10 or 12% SDS-polyacrylamide gels. The separated proteins were transferred onto PVDF membranes using a wet protein transfer system (Bio-Rad, West Berkeley, CA, USA) for 1.5 h in an icebox. After blocking with 5% nonfat dry milk, the membranes were incubated overnight at 4 °C with the primary antibodies listed in Table 1. Subsequently, the membranes were incubated with specific HRP-conjugated secondary antibodies at 37 °C for 1 h. Ultimately, the immunoreactive signals were visualized using an electrochemiluminescence system (Bio-Rad, West Berkeley, CA, USA), and the relative intensities of the bands were quantified using Image Lab software (Bio-Rad, West Berkeley, CA, USA). According to the experiment, β-actin, β-tubulin, GAPDH, and COX IV were selected as internal controls.

### RNA extraction and real-time quantitative PCR (RT-qPCR) assay

Total RNA isolation and real-time PCR were conducted as described in our previous study [27]. Briefly, BMSCs were treated as indicated. Total RNA was isolated from the cells with TRIzol reagent. After isolation, the RNA quality was assessed on a NanoDrop2000 spectrophotometer (Thermo Scientific, Waltham, MA, USA) according to the manufacturer’s instructions. Absorbance ratios (260/280) ranging from 1.8–2.0 indicated pure RNA samples. Reverse transcription was conducted with oligo-dT to prime the reverse transcription reaction, and the *apoptotic protease activating factor-1 (Apaf-1)* primers used to amplify cDNA are listed in Table 2. Real-time PCR cycling was carried out using a Bio-Rad CFX96 Real-Time System (Bio-Rad, West Berkeley, CA, USA). The PCR mixture consisted of 10 μL of 2× TB Green Premix Ex Taq II, 0.8 μL of both sense and antisense primers, 2.0 μL of sample cDNA solution, and distilled water to a final volume of 20 μL. The cycling program was as follows: 95 °C for 30 s and 40 cycles of 95 °C for 5 s and 60 °C for 30 s, with a melting curve from 65 to 95 °C to ensure amplification of a single product. The mRNA levels were calculated using the 2^−ΔΔCt^ method (relative fold change). *GAPDH* was amplified as an internal control.

### Statistical analysis

The results are expressed as the mean ± standard deviation (SD) unless otherwise specified. Skewness and kurtosis were used to describe the data distribution. Then, the Shapiro-Wilk test was further applied to evaluate normality. Group comparisons were performed using one-way ANOVA followed by the LSD test (SPSS 13.0, SPSS Inc., Chicago, IL, USA). All in vitro and imaging studies were performed in a blinded manner. *p* < 0.05 was considered to indicate a statistically significant difference.

## Results

### Characterization of mouse BMSCs

**Fig. 1:**
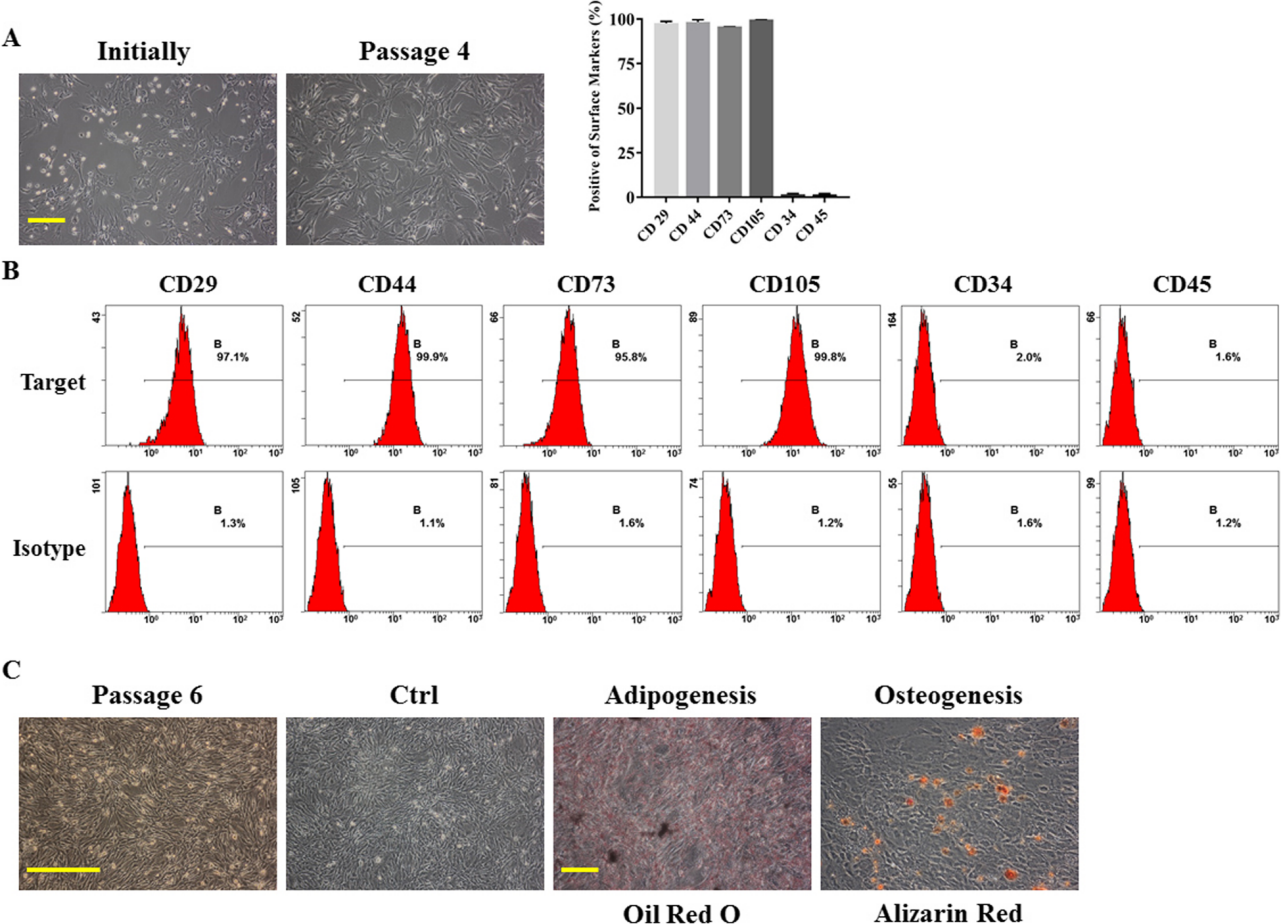
Characterization of mouse BMSCs. **a** The spindle-shaped morphology of bone marrow cells that appeared at day 7. More confluent BMSCs were observed at P4 under light microscopy. **b** The expression of surface marker molecules in BMSCs (P4) was analyzed and quantified by flow cytometry, and the cells were positive for MSC markers (CD29, CD44, CD73, and CD105) and negative for hematopoietic antigens (CD34 and CD45). Additionally, nonspecific fluorescence was determined by incubation with isotype-matched anti-mouse monoclonal antibodies. **c** Representative images of BMSC morphology (P6) and adipogenic and osteogenic differentiation visualized by Oil Red O and Alizarin Red staining, respectively. Cells treated with BM served as a control group. Scale bar = 100 μm. Data are presented as the mean ± SD (*n* = 3 independent experiments). Images are shown at the original magnification

When BMSCs were cultured, the cells began to attach 6–8 h after the tibial and femoral marrow compartments were flushed; this process was used to separate the BMSCs from contaminating hematopoietic cells. Morphologically, the adherent cells initially showed a polygonal appearance and then grew rapidly and became spindle- or fibroblast-shaped (Fig. 1a). As shown by flow cytometry analysis, most adherent cells highly expressed CD29, CD44, CD73, and CD105 (MSC markers) and were nearly negative for CD34 and CD45 (hematopoietic antigens) (Fig. 1b). These results indicated that BMSCs separated from the bone marrow of mouse long bones were of high purity. The multipotent differentiation capacity of the BMSCs was also analyzed. After 3 weeks of adipogenic induction, the cells were fixed with 4% paraformaldehyde at room temperature for 30 min and incubated with Oil Red O solution. Many oil droplets were observed in the cytoplasm. After approximately 4 weeks of osteogenic induction, mineralized nodules were visualized by Alizarin Red staining (Fig. 1c). These morphological changes indicated that the cells had complete multipotent differentiation capacity.

### Effect of H_2_O_2_ on BMSC apoptosis

**Fig. 2:**
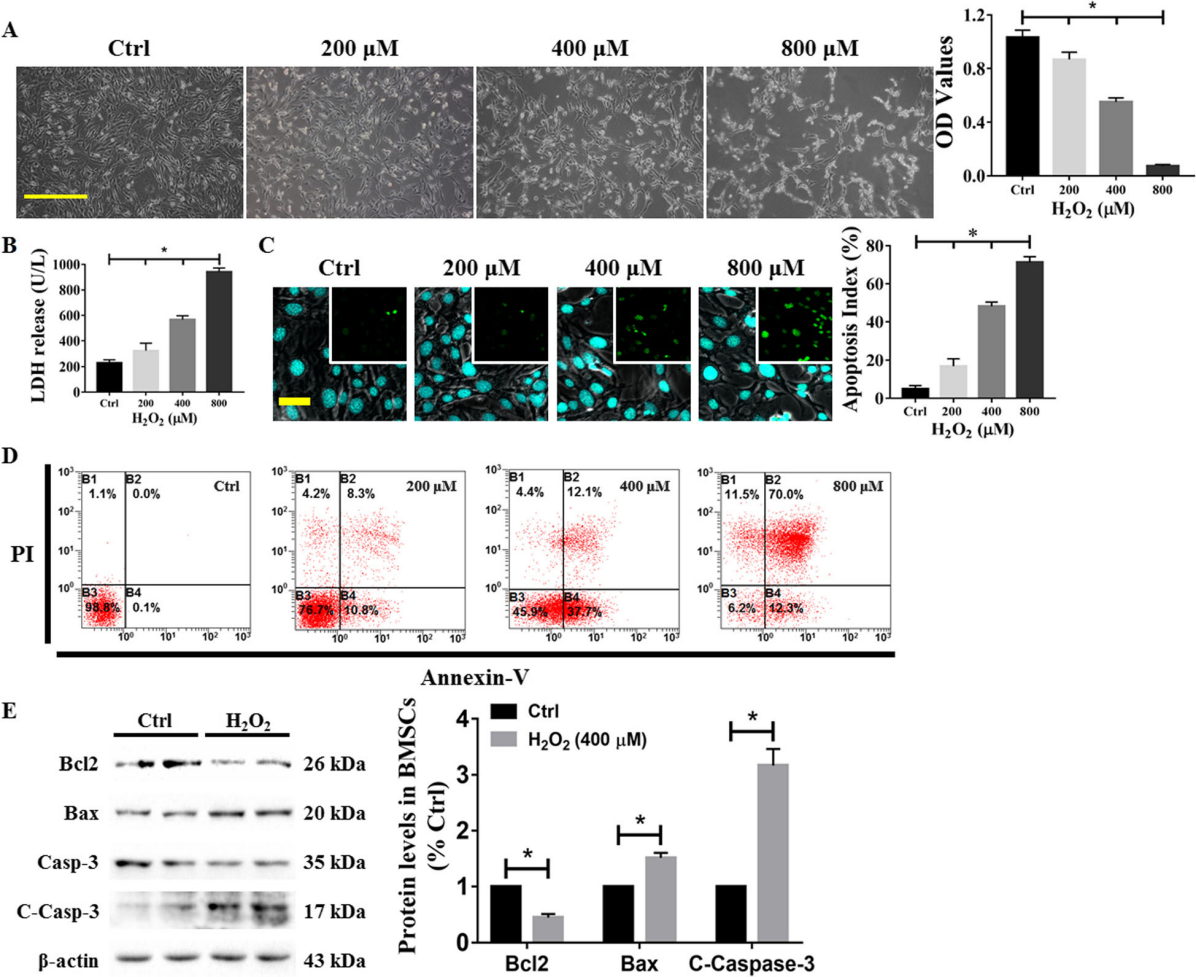
Effect of H_2_O_2_ on apoptosis in BMSCs. BMSCs were injured with H_2_O_2_ at different concentrations from 200 to 800 μM for 24 h, and the **a** cell viability (scale bar = 100 μm), **b** LDH concentration, and **c** degree of apoptosis indicated by TUNEL^+^ (scale bar = 50 μm) and **d** Annexin V^+^/PI^+^-labeled cells were then determined. Nuclei were labeled with DAPI. Moreover, BMSCs were exposed to H_2_O_2_ for 24 h, and cell lysates were collected and subjected to western blot detection of the Bcl2, Bax, and C-Casp-3 proteins, with normalization to β-actin. Cells treated with PBS served as a control group. Values are expressed as the mean ± SD (*n* = 3 independent experiments). ^*^p < 0.05 compared with the control group. One-way ANOVA followed by the LSD test was used to analyze the significance of differences. Morphological images are shown at the original magnification. C-Casp-3, cleaved Caspase-3; LDH, lactate dehydrogenase; OD, optical density

To construct a model of oxidative stress, we treated BMSCs with different concentrations of H_2_O_2_ ranging from 200 to 800 μM for 24 h in 96-well plates. The CCK-8 assay was performed, and the OD values were analyzed at 450 nm. The results showed that the viability of BMSCs decreased with increasing concentrations of H_2_O_2_, and the IC50 value was approximately 400 μM (Fig. 2a). LDH, which is retained in the cytoplasm of viable cells with intact plasma membranes and released from cells after membrane damage, is considered an important indicator of necrosis [28]. Spectrophotometric analysis demonstrated that the LDH level was significantly increased in the medium of cells treated with H_2_O_2_, especially those treated with 800 μM H_2_O_2_ (Fig. 2b). Additionally, TUNEL staining, which is used to detect apoptosis, revealed that H_2_O_2_ induced a significant increase in green fluorescence (Fig. 2c); this was also verified by Annexin V and PI labeling of the cells (Fig. 2d). These results indicated that H_2_O_2_ obviously increased the cell apoptotic index in a dose-dependent manner. Moreover, with increasing H_2_O_2_ concentrations, the cells showed irreversible damage, which was manifested by the development of early to late apoptosis (Fig. 2d). The cells were subsequently harvested for western blot analysis. As expected, increasing H_2_O_2_ concentrations were associated with BMSC apoptosis, as evidenced by a decrease in the expression of the antiapoptotic protein Bcl2 and increases in the expression of the proapoptotic protein Bax and the apoptosis effector cleaved Caspase-3 (C-Casp-3) (Fig. 2e).

### Effects of H_2_O_2_ on mitochondrial dysfunction, ER stress, and AMPK signaling in BMSCs

**Fig. 3:**
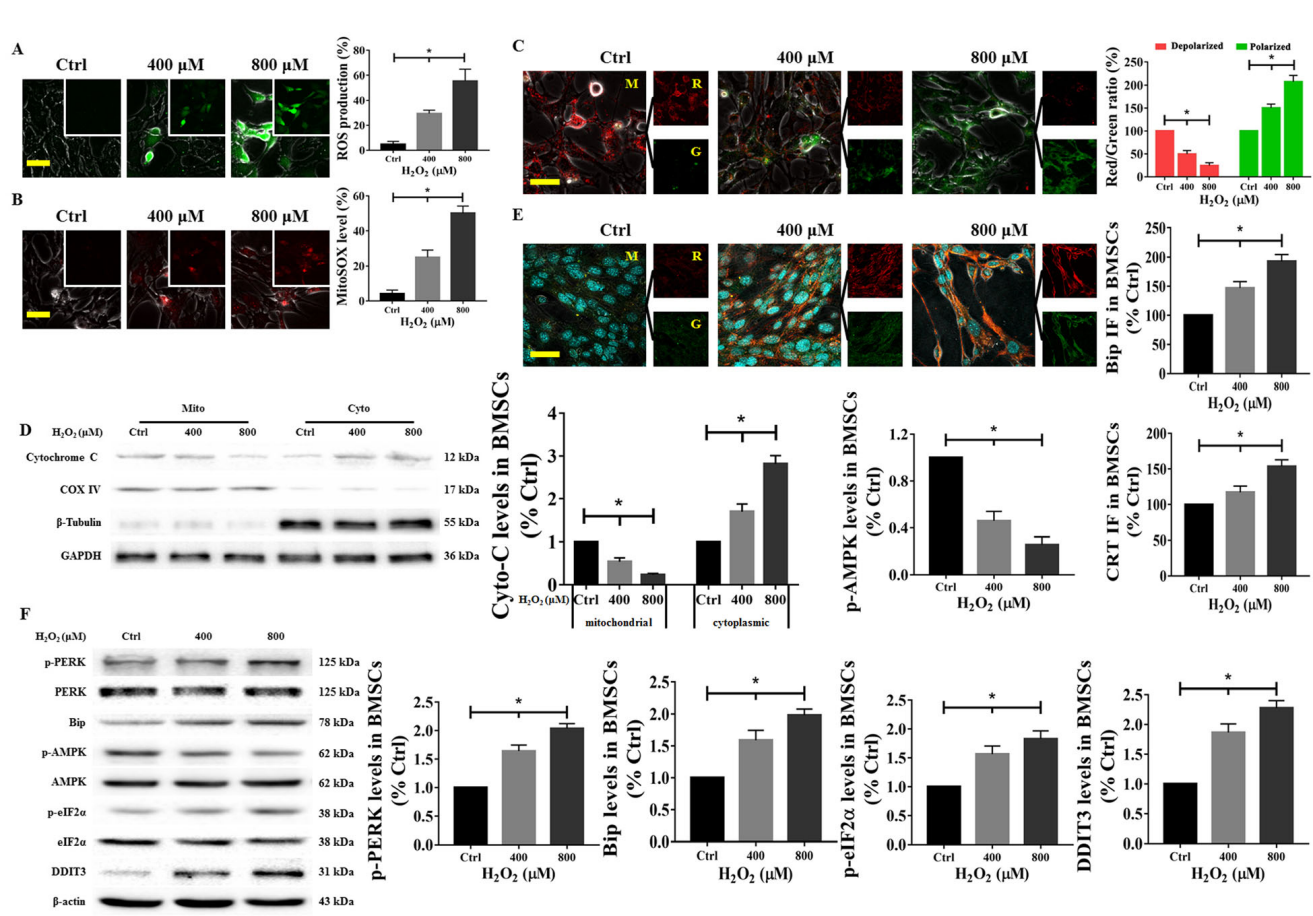
Effect of H_2_O_2_ on mitochondrial dysfunction, ER stress, and AMPK signaling in BMSCs. BMSCs were treated with H_2_O_2_ at different concentrations from 200 to 800 μM for 24 h, and then (**a**) ROS production, (**b**) mitochondrial superoxide levels, and (**c**) MMP levels were analyzed. **d** Western blot measurement and quantification of Cyto-C translocation immunostaining in BMSCs after H_2_O_2_ injury for 24 h. **e** Representative images of Bip (red) and CRT (green) immunostaining in BMSCs treated with H_2_O_2_ for 24 h. Nuclei are labeled with DAPI. **f** After treatment with H_2_O_2_, the levels of p-AMPK and proteins related to ER stress, such as p-PERK, Bip, p-eIF2α, and DDIT3, were determined using western blotting in BMSCs, with normalization to the values of β-actin. Cells treated with PBS served as a control group. Values are expressed as the mean ± SD (*n* = 3 independent experiments). ^*^p < 0.05 compared with the control group. One-way ANOVA followed by the LSD test was used to analyze the significance of differences. Scale bar = 50 μm. CRT, calreticulin; Cyto-C, Cytochrome C; ROS, reactive oxygen species

ROS are important mediators of H_2_O_2_-induced cell death and are mainly produced in mitochondria [11, 29]. After being treated with different concentrations of H_2_O_2_, BMSCs were stained with DCFH-DA solution. DCFH-DA can be hydrolyzed to DCFH, which is further oxidized to fluorescent DCF by ROS in the cytoplasm. As shown in Fig. 3a, H_2_O_2_ stimulation resulted in enhanced green fluorescence in BMSCs, which indicated an increase in ROS production. Additionally, the MitoSOX™ Red mitochondrial superoxide indicator, a novel fluorogenic dye that is used for the highly selective detection of superoxides in the mitochondria of live cells and that is readily oxidized by superoxides but not by other ROS- or reactive nitrogen species (RNS)-generating systems, was used. Our results showed that H_2_O_2_-induced red fluorescence rapidly increased in BMSCs, and this finding was consistent with the trend of ROS being present throughout the cell (Fig. 3b).

Mitochondrial bioenergetics is closely associated with a normal MMP level, which is important for ROS scavenging and cytoprotection against apoptosis induced by excessive ROS [30, 31]. The fluorescent lipophilic cation JC-1 is an MMP-sensitive dye that aggregates in the mitochondrial matrix. It exhibits red fluorescence in healthy cells and emits green fluorescence as a monomer [31]. As shown in Fig. 3c, the MMP, as assessed by staining with JC-1, decreased in a dose-dependent manner in response to H_2_O_2_ treatment. Moreover, the MMP is closely related to the integrity of the mitochondrial membrane [32]. When the mitochondrial outer membrane is disrupted, intermembrane space proteins, notably Cyto-C, are released into the cytosol [32]. Accordingly, western blotting was performed, and it demonstrated that Cyto-C translocated from the mitochondria to the cytoplasm; COX VI and β-tubulin served as loading controls, indicating successful mitochondrial isolation (Fig. 3d).

ROS have emerged as crucial regulators of ER function and AMPK activation. Therefore, we investigated the effects of H_2_O_2_ on these two pivotal processes in BMSCs. Immunofluorescence revealed increases in red (indicating Bip expression) and green (indicating expression of CRT, a marker of the ER lumen [33]) fluorescence intensities (Fig. 3e). Additionally, when the BMSCs were harvested, the levels of proteins related to ER stress, such as phospho (p)-PERK, Bip, p-eIF2α, and DDIT3, were rapidly increased in the H_2_O_2_-treated group. Simultaneously, the levels of p-AMPK were significantly decreased by H_2_O_2_ treatment (Fig. 3f). This evidence suggests that ROS produced in mitochondria directly or indirectly affect ER homeostasis and AMPK activity, as reported in previous studies [11–13].

### Effect of melatonin on the cell biology of BMSCs

**Fig. 4:**
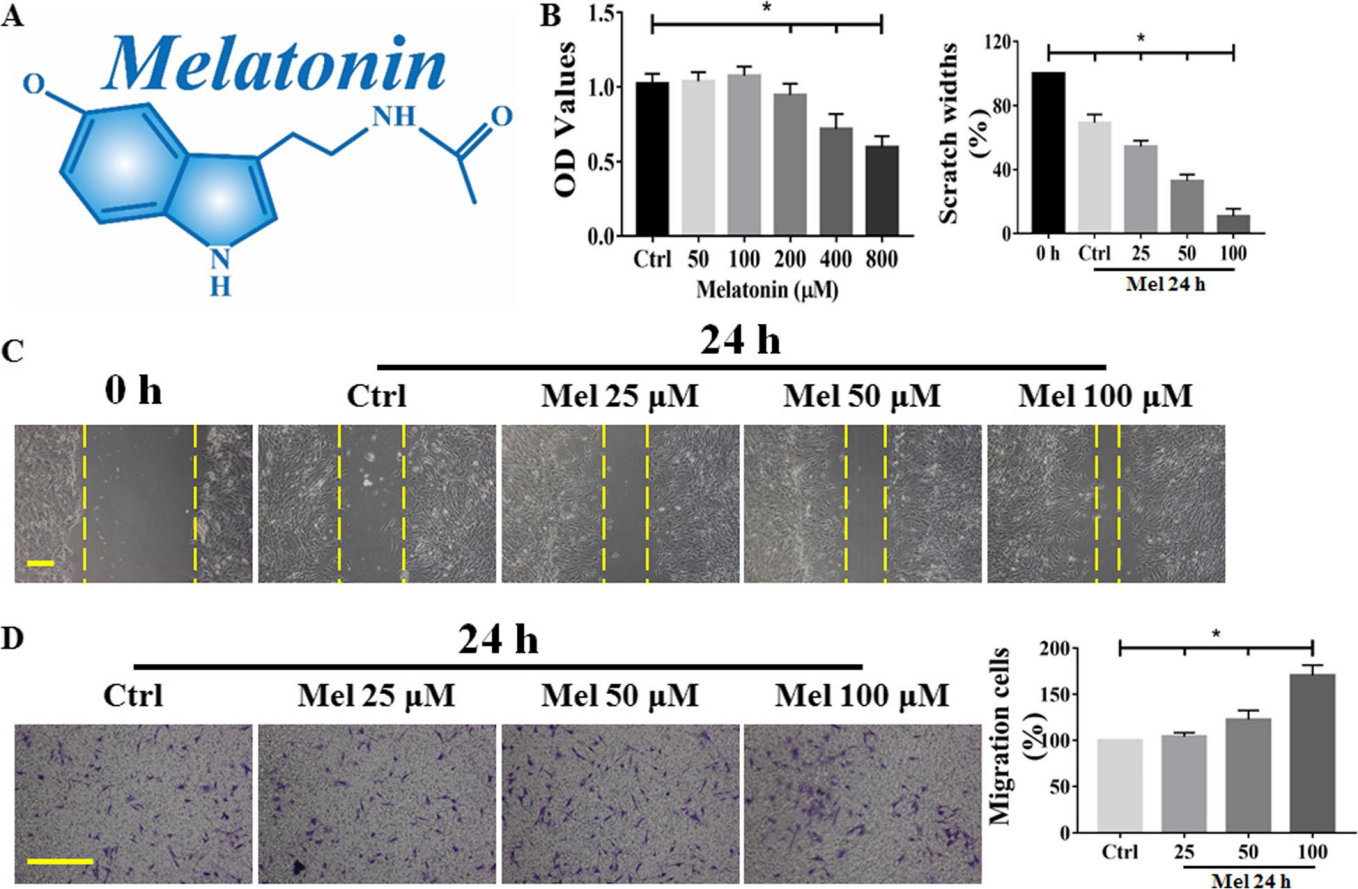
Effect of melatonin on cell biological behaviors in BMSCs. BMSCs were incubated with different concentrations of melatonin for 24 h. BMSCs seeded in 96-well plates were treated with melatonin (ranging from 50 to 800 μM) for 24 h. **a** Chemical structure of melatonin (Mel). **b** Cell viability was determined by a CCK-8 assay. Scratch migration (**c**) and Transwell chemotaxis assays (**d**) were performed for 24 h with melatonin treatment. Scale bar = 100 μm. Cells treated with PBS served as a control group. Values are expressed as the mean ± SD (*n* = 3 independent experiments). ^*^p < 0.05 compared with the control group. One-way ANOVA followed by the LSD test was used to analyze the significance of differences. Images are shown at the original magnification. Mel, melatonin; OD, optical density

To determine whether melatonin (the molecular structure of which is shown in Fig. 4a) could directly or indirectly regulate cell biological characteristics, we examined the cytoactivity and migration of BMSCs. First, the cytotoxicity of different concentrations of melatonin was analyzed. BMSCs seeded in 96-well plates were treated with melatonin (ranging from 50 to 800 μM) for 24 h, and cell viability was detected by the CCK-8 assay. The results showed that the administration of melatonin at a concentration lower than 100 μM had almost no effect, while the OD values decreased by 10% upon treatment with 200 μM melatonin (Fig. 4b). Therefore, the maximal concentration of melatonin used in our current study was 100 μM. In the next step, the effect of melatonin on BMSC migration was evaluated by assessing Transwell chemotaxis and performing a wound-healing assay. As evidenced by the wound-healing assay, treatment with various concentrations of melatonin decreased the distance between BMSCs on the two sides of a scratch created by a 200-μL pipet tip after 24 h (Fig. 4c). Moreover, as illustrated in Fig. 4d, the two-chamber migration assay and crystal violet staining showed that melatonin treatment for 24 h increased the number of cells on the bottom of the membrane insert. These data suggested that melatonin could promote BMSC migration and that melatonin could boost the biological behaviors of BMSCs to a certain extent.

### Effect of melatonin on cell damage induced by H_2_O_2_ in BMSCs

**Fig. 5:**
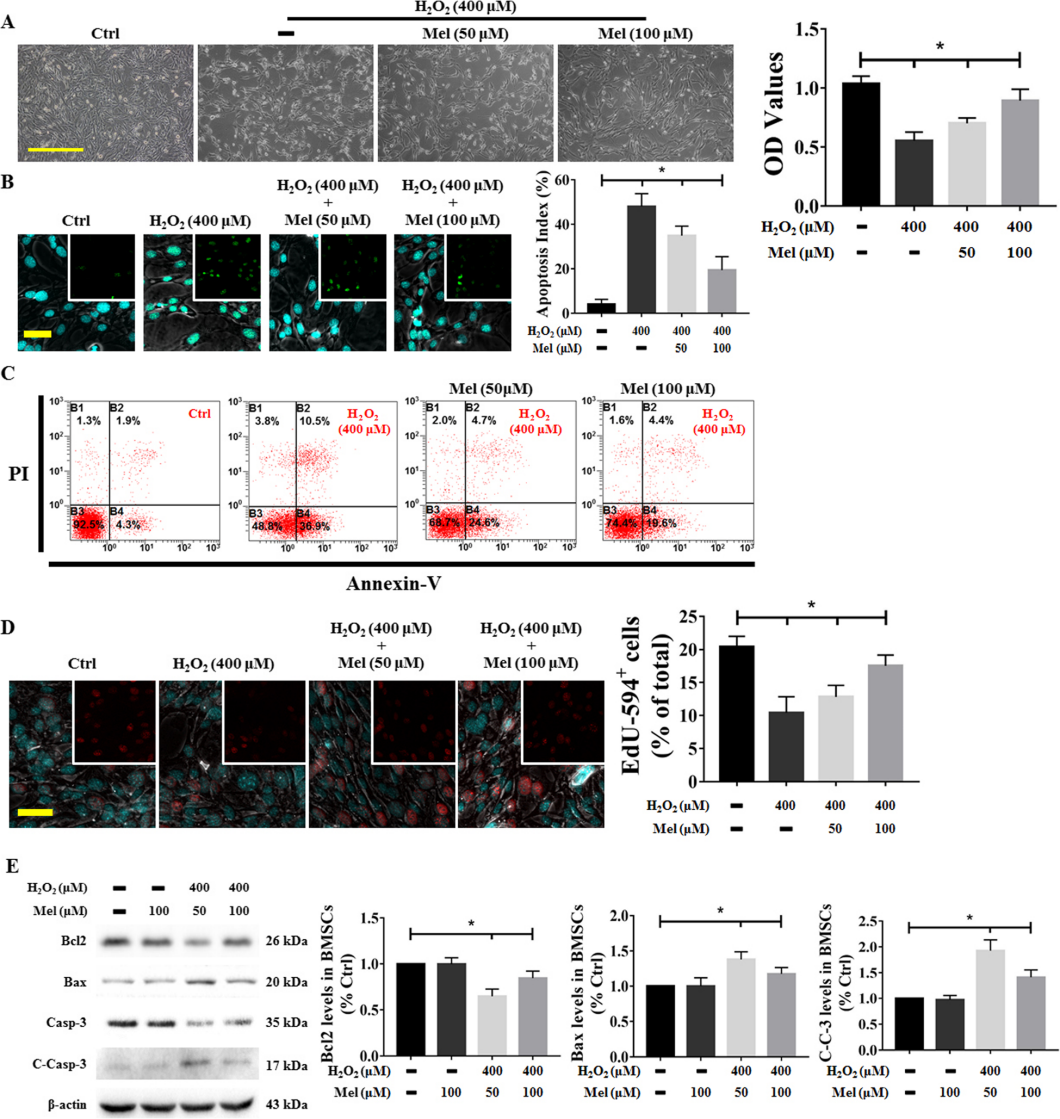
Effect of melatonin on H_2_O_2_-induced cell damage in BMSCs. BMSCs pretreated with melatonin at different concentrations (50 and 100 μM) for 6 h were exposed to 400 μM H_2_O_2_ for another 24 h. The **a** cell viability (scale bar = 100 μm), **b** TUNEL^+^ cells (scale bar = 50 μm), and **c** Annexin V^+^/PI^+^-labeled cells are presented. Nuclei were labeled with DAPI. **d** Representative images of EdU-594^+^ cells are shown on microscope slides. Nuclei were labeled with DAPI. **e** Apoptosis regulatory elements, including the Bcl2, Bax, and cleaved Caspase-3 (C-Casp-3) proteins, were determined using western blotting. Cells treated with PBS served as a control group. Values are expressed as the mean ± SD (*n* = 3 independent experiments). ^*^p < 0.05 compared with the control group. One-way ANOVA followed by the LSD test was used to analyze the significance of differences. Images are shown at the original magnification. C-Casp-3 (C-C-3), cleaved Caspase-3; Mel, melatonin; OD, optical density

To investigate the protective effect of melatonin, BMSCs pretreated with different concentrations of melatonin (50 and 100 μM) for 6 h were exposed to 400 μM H_2_O_2_ for another 24 h, and their viability was analyzed by the CCK-8 assay. The cell viability of the H_2_O_2_ group was significantly decreased compared with that of the control group, and a gradual increase in the melatonin concentration promoted cell survival, as evidenced by a more typical cell morphology and higher OD values (Fig. 5a). Additionally, H_2_O_2_ insult increased the apoptotic index, and melatonin treatment markedly reduced H_2_O_2_-induced apoptosis (Fig. 5b). The Annexin V-FITC/PI staining results revealed that H_2_O_2_ injury caused a significant increase in the incidence of apoptosis, while melatonin administration decreased the apoptosis rate (Fig. 5c). In addition, BMSCs were labeled with EdU-Alexa Fluor 594 to determine whether melatonin could enhance cell proliferation. The immunostaining results revealed an increase in the number of EdU-594^+^ BMSCs after treatment with melatonin compared with the H_2_O_2_ treatment (Fig. 5d). Consistent with the above results, melatonin not only increased Bcl2 expression but also decreased Bax and C-Casp-3 expression after oxidative stress injury, providing molecular evidence that the protective effect of melatonin against H_2_O_2_ damage might be due to inhibition of apoptosis (Fig. 5e).

Moreover, NAC (a free radical scavenger that acts as the precursor of glutathione and as a scavenger of free radicals [34]) was used to explore whether the mitochondria-dependent protective effects of melatonin on cell apoptosis were due to the inhibition of ROS production. As indicated in Supplementary Figure 1, BMSCs were preprocessed with NAC (1 mM) in the absence or presence of melatonin (100 μM) for 6 h and then exposed to 400 μM H_2_O_2_ for another 24 h. Cotreatment with NAC and melatonin further promoted cell viability (Supplementary Figure 1A) and inhibited ROS production (Supplementary Figure 1B). Moreover, the apoptotic index and C-Casp-3 fluorescence were significantly decreased in the NAC + melatonin group compared with the melatonin group (Supplementary Figure 1C-1E). The RT-qPCR results showed that BMSCs treated with 100 μM H_2_O_2_ had significantly higher expression of *Apaf-1* than did the control group, while the expression of this factor was reduced after melatonin treatment, with reduced expression in the NAC + melatonin group (Supplementary Figure 1F). Additionally, NAC and melatonin treatment obviously increased the protein expression of Bcl2 and decreased the levels of Bax, C-Casp-3, and Apaf-1 during H_2_O_2_-induced injury (Supplementary Figure 1G).

### Effect of melatonin on mitochondrial function, ER stress, and AMPK signaling in response to H_2_O_2_-induced BMSC insult

**Fig. 6:**
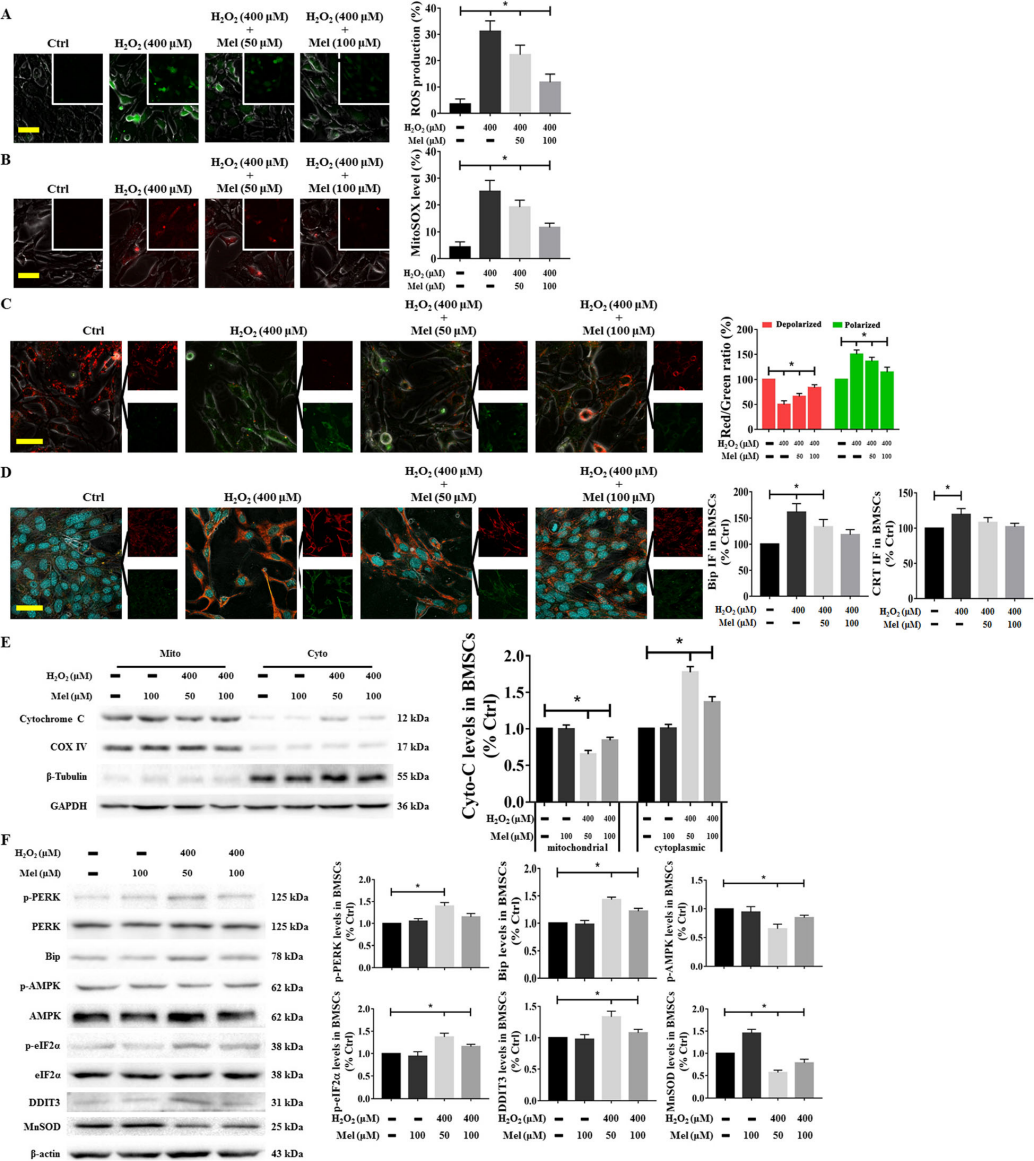
Effect of melatonin on mitochondrial function, ER stress, and AMPK signaling in response to H_2_O_2_-induced insults in BMSCs. BMSCs pretreated with melatonin at different concentrations (50 and 100 μM) for 6 h were exposed to 400 μM H_2_O_2_ for another 24 h. **a** ROS production, **b** mitochondrial superoxide levels, and **c** MMP levels were analyzed. **d** After pretreatment with melatonin at different concentrations, the cells subjected to H_2_O_2_ were stained with primary antibodies against Bip and CRT. Representative images of immunostaining in BMSCs are shown. Nuclei were labeled with DAPI. **e** Western blotting was used to measure and quantify the expression of cytochrome C (Cyto-C) translocation immunostaining in BMSCs. **f** After treatment with melatonin and H_2_O_2_, the levels of p-AMPK and proteins related to ER stress, such as p-PERK, Bip, p-eIF2α, and DDIT3, were determined using western blotting in BMSCs, and the values were normalized to that of β-actin. Cells treated with PBS served as a control group. Values are expressed as the mean ± SD (*n* = 3 independent experiments). ^*^p < 0.05 compared with the control group. One-way ANOVA followed by the LSD test was used to analyze the significance of differences. Scale bar = 50 μm. CRT, calreticulin; Cyto-C, Cytochrome C; Mel, melatonin; ROS, reactive oxygen species

As mentioned above, mitochondrial dysfunction, ER stress, and AMPK inactivation are three important regulatory mechanisms for oxidative stress injury. Theoretically, restoring mitochondrial function, inhibiting ER stress, or increasing AMPK activity would have a beneficial effect on the ability of BMSCs to withstand H_2_O_2_-induced damage. Considering the ability of melatonin to scavenge oxygen-free radicals, we next explored the potential mechanism by which it protects BMSCs from H_2_O_2_-induced apoptosis. As shown in Fig. 6a–c, H_2_O_2_ accelerated whole-cell and mitochondrial superoxide production and depolarization of the mitochondrial membrane, while pretreatment with melatonin significantly attenuated the accumulation of ROS and increased the MMP. In addition, melatonin reduced the expression of Bip and CRT after H_2_O_2_ insult (Fig. 6d), as indicated by weakened fluorescence intensities (red is indicated as Bip and green is indicated as CRT). The western blot analysis also demonstrated that treatment with melatonin prevented the translocation of Cyto-C to the cytosol and increased the levels of ER stress factors (p-PERK, Bip, p-eIF2α, and DDIT3) (Fig. 6e and f). Furthermore, AMPK activity was also enhanced by melatonin in H_2_O_2_-treated BMSCs, as evidenced by the elevation of p-AMPK (Fig. 6f).

### Regulatory effects of activated AMPK and ER stress on melatonin-mediated cellular protection against H_2_O_2_ damage

**Fig. 7:**
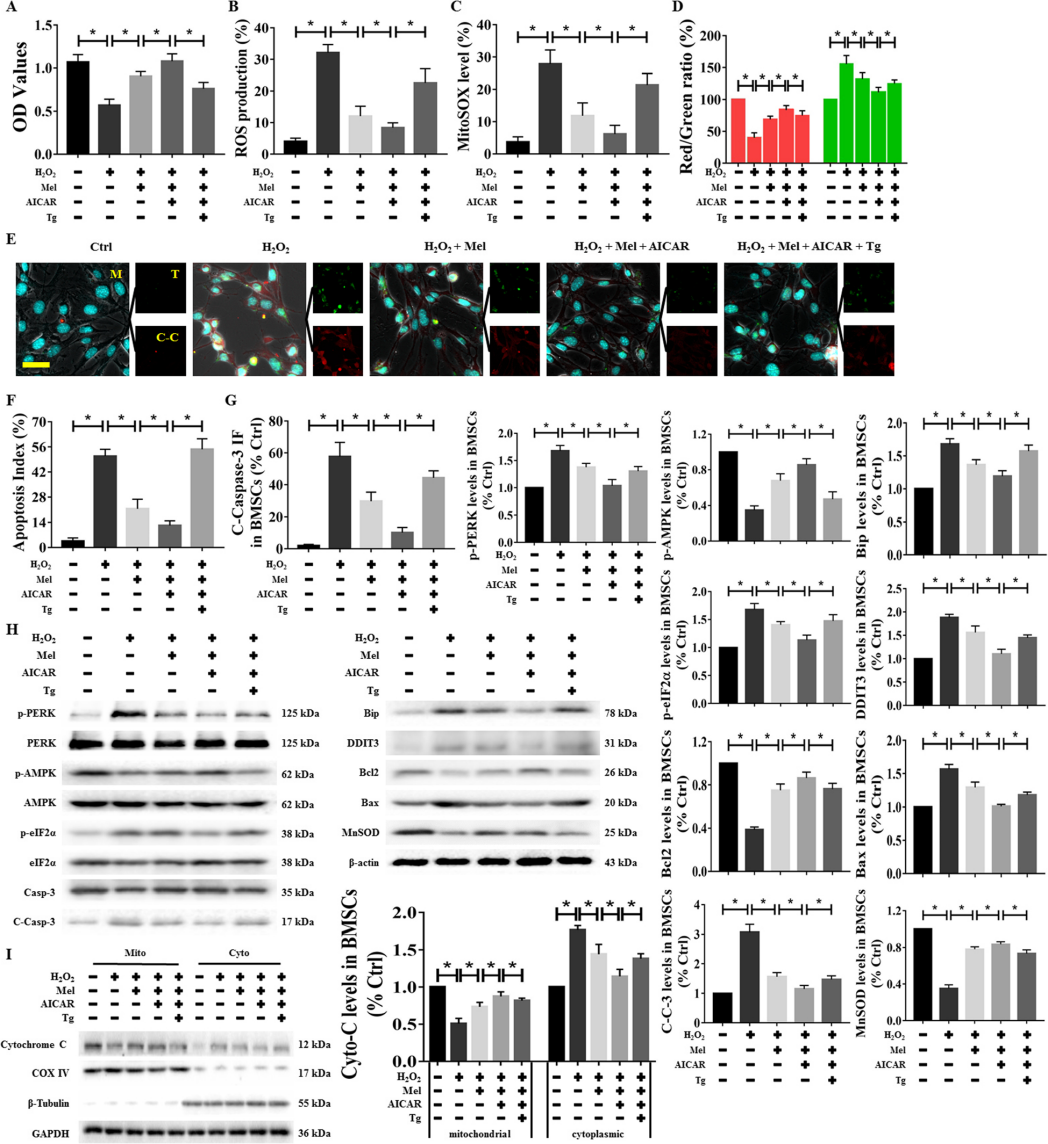
Regulatory effects of activated AMPK or ER stress on melatonin-mediated cellular protection against H_2_O_2_-induced damage. BMSCs were treated using the indicated agents for the indicated times to preactivate AMPK and ER stress. **a** Cell viability, **b** ROS production, **c** mitochondrial superoxide levels, and **d** MMP levels are shown. **e**–**g** The TUNEL assay and C-Casp-3 immunofluorescence staining were conducted. The percentage of TUNEL^+^ cells and the C-Casp-3 fluorescence intensity were used to determine the degree of apoptosis. Nuclei were labeled with DAPI. **h**, **i** Western blots were used to measure and quantify the expression levels of p-AMPK, ER stress factors, apoptosis-related parameters, and mitochondrial function markers in BMSCs. p-PERK, Bip, p-eIF2α, and DDIT3 were the ER stress factors. Bcl2, Bax, and C-Casp-3 were the apoptosis-related parameters. Cyto-C served as the marker of mitochondrial function. Cells treated with PBS served as a control group. Values are expressed as the mean ± SD (*n* = 3 independent experiments). ^*^p < 0.05 compared with different groups. One-way ANOVA followed by the LSD test was used to analyze the significance of differences. Scale bar = 50 μm. AICAR, acadesine; C-Casp-3 (C-C-3, C-C), cleaved Caspase-3; Cyto-C, cytochrome C; Mel, melatonin; TG, thapsigargin; OD, optical density; ROS, reactive oxygen species

To investigate the roles of AMPK and ER stress in melatonin-induced cellular protection against H_2_O_2_ exposure, we further upregulated the expression of AMPK and induced ER stress with AICAR and TG, respectively. The cells were processed as follows: ① For AMPKα activation, BMSCs were incubated with AICAR dissolved in 0.1% DMSO in the presence of melatonin for 6 h (Supplementary Figure 2Aa) and ② cells were co-incubated with AICAR, TG, and melatonin to induce ER stress in advance (Supplementary Figure 2Ab) and then washed with PBS and cultured with 400 μM H_2_O_2_ for another 24 h. The results presented in Supplementary Figure 2B show that treatment with these two small-molecule agents at concentrations less than 500 μM for 24 h marginally inhibited the viability of BMSCs. Additionally, at these concentrations, the expression levels of p-AMPK and p-PERK protein were significantly promoted (Supplementary Figure 2C). We found that pretreatment with AICAR significantly promoted the protective effects of melatonin on cell viability (Fig. 7a), ROS production (Fig. 7b and Supplementary Figure 3A), mitochondrial superoxide levels (Fig. 7c and Supplementary Figure 3B), and the MMP (Fig. 7d and Supplementary Figure 3C) in BMSCs exposed to H_2_O_2_-induced injury. Moreover, cell apoptosis was evaluated by C-Casp-3 immunofluorescence followed by TUNEL staining, as described previously. Pretreatment with AICAR and melatonin further decreased the green (TUNEL^+^ cells) and red (C-Casp-3-labeled cells) fluorescence intensities, which indicated that the combination of these agents had a stronger antiapoptotic effect than did either agent alone (Fig. 7e–g). Additionally, AICAR treatment markedly enhanced the regulatory effect of melatonin on proteins of interest, including p-AMPK, ER stress markers (p-PERK, Bip, p-eIF2α, and DDIT3), apoptosis regulatory proteins (C-Casp-3, Bcl2, and Bax), and mitochondrial oxidative stress-related proteins (Cyto-C and MnSOD) (Fig. 7h, i). Conversely, compared with AICAR and melatonin treatment, the addition of TG reduced BMSC viability (Fig. 7a), ROS production (Fig. 7b and Supplementary Figure 3A), mitochondrial superoxide levels (Fig. 7c and Supplementary Figure 3B), and the MMP (Fig. 7d and Supplementary Figure 3C) during H_2_O_2_-induced injury. As expected, the regulatory effects of AICAR and melatonin treatment on the apoptotic index and the abovementioned proteins were abolished after TG administration (Fig. 7e–i).

### Regulatory effects of inactivated AMPK and ER stress on melatonin-mediated cellular protection against H_2_O_2_-induced damage

**Fig. 8:**
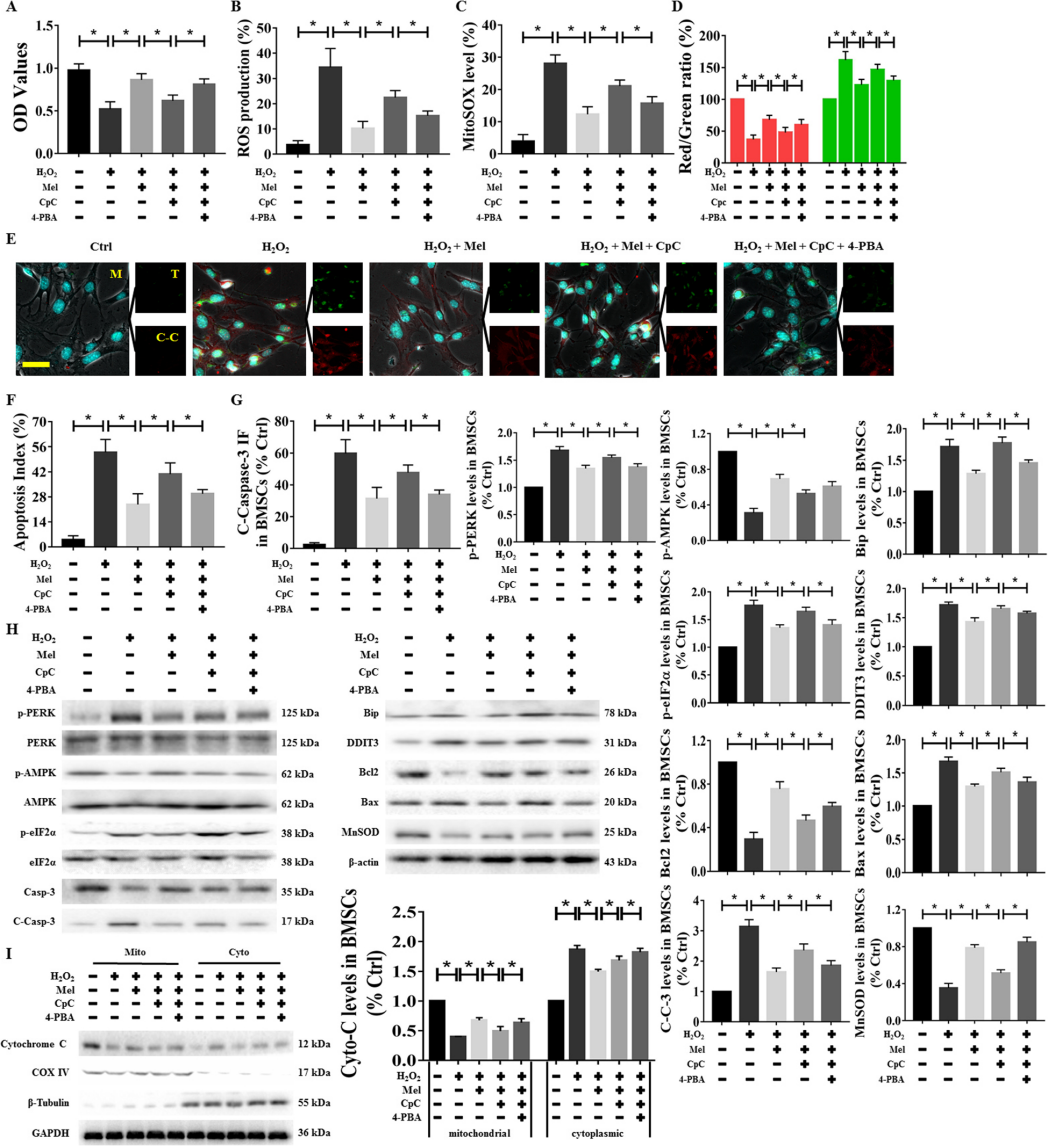
Regulatory effects of inactivated AMPK or ER stress on melatonin-mediated cellular protection against H_2_O_2_ damage. BMSCs were treated using the indicated agents for the indicated times to preinhibit AMPK and ER stress. **a** Cell viability, **b** ROS production, **c** mitochondrial superoxide levels, and **d** MMP levels are shown. **e**–**g** The TUNEL assay and C-Casp-3 immunofluorescence staining were conducted. The percentage of TUNEL^+^ cells and C-Casp-3 fluorescence intensity were used to determine the degree of apoptosis. Nuclei were labeled with DAPI. **h**, **i** Western blotting was used to measure and quantify the expression levels of p-AMPK, ER stress factors, apoptosis parameters, and mitochondrial function markers in BMSCs. p-PERK, Bip, p-eIF2α, and DDIT3 were the ER stress factors. Bcl2, Bax, and C-Casp-3 were the apoptosis parameters. Cyto-C served as the marker of mitochondrial function. Cells treated with PBS served as a control group. Values are expressed as the mean ± SD (*n* = 3 independent experiments). ^*^p < 0.05 compared with different groups. One-way ANOVA followed by the LSD test was used to analyze the significance of differences. Scale bar = 50 μm. C-Casp-3 (C-C-3, C-C), cleaved Caspase-3; CpC, compound C dihydrochloride; Cyto-C, cytochrome C; Mel, melatonin; OD, optical density; ROS, reactive oxygen species; 4-PBA, 4-phenylbutyric acid

To further analyze the relationship between AMPK and ER stress in the melatonin-mediated antiapoptotic effects during H_2_O_2_-induced injury, the negative regulatory reagents CpC and 4-PBA were used to downregulate their respective targets. As with agonist application, safe concentrations were first identified and then applied to cell cultures for the indicated periods. The CCK-8 results demonstrated that CpC and 4-PBA started to inhibit cell viability at concentrations of 6 μM and 200 μM, respectively (Supplementary Figure 2D), and the expression of their target proteins was obviously inhibited at these concentrations (Supplementary Figure 2E). Therefore, in addition to being treated with CpC (4 μM) and melatonin (100 μM), the cells were coincubated with CpC (4 μM), 4-PBA (100 μM), and melatonin (100 μM) for 6 h and then cultured with 400 μM H_2_O_2_ for another 24 h (Supplementary Figure 2Ac and Ad). Melatonin treatment obviously increased cellular activity and the MMP and decreased the ROS production, Mito-SOX fluorescence, and cellular apoptosis, and these effects were reversed by CpC pretreatment (Fig. 8a–g and Supplementary Figure 4A-4C). Moreover, after CpC treatment, the melatonin-mediated downregulation of proteins (p-PERK, Bip, p-eIF2α, DDIT3, C-Casp-3, and Bcl2), the translocation of Cyto-C, and the upregulation of certain proteins (Bax and MnSOD) during H_2_O_2_-induced injury were reduced (Fig. 8h, i). However, treatment of the BMSCs with 4-PBA partially normalized cell functions and alleviated oxidative stress levels and apoptosis (Fig. 8a–i and Supplementary Figure 4A-4C). Additionally, AMPK siRNA and DDIT3 siRNA reagents did not affect cell activity despite effectively reducing the levels of their respective target proteins (Supplementary Figure 2F). After pretreatment with AMPK siRNA, the melatonin-mediated upregulation of cytoactivity during H_2_O_2_-induced injury was reduced, while the administration of DDIT3 siRNA promoted the cytoprotective effect of melatonin in BMSCs (Supplementary Figure 2Ae and G). These results provide further evidence that melatonin exerts cell-protective effects through activation of AMPK and inhibition of the ER stress pathway, as AMPK-induced increases in the protective effect of melatonin contribute to a partial reduction of ER stress in BMSCs.

## Discussion

The molecular pathways responsible for the beneficial effects of BMSC transplantation as a cell-based strategy are still not fully understood, although stem cells have the potential to be used for clinical applications. Understanding the regulatory mechanisms by which BMSCs promote colonization, especially in the oxidative microenvironment after injury, will greatly contribute to improving their clinical efficacy. In this study, we first established a canonical oxidative stress injury model with H_2_O_2_, which is a classical signaling molecule that is commonly used to establish models of exogenous oxidative stress because of its unique biochemical properties, such as a relatively long half-life and solubility in both lipids and aqueous media [35, 36]. Then, we developed a novel strategy to preserve the biological function of BMSCs based on the specific small bioactive substance melatonin. Here, we report that treatment with H_2_O_2_ inhibited cell viability and induced BMSC apoptosis. From a molecular perspective, H_2_O_2_ insult promoted the accumulation of ROS, disrupted mitochondrial energy metabolism, promoted oxidative stress, suppressed cytoprotective signals, and initiated ER stress in BMSCs. Importantly, H_2_O_2_-mediated cell apoptosis, mitochondrial injury, and ER stress were prevented by melatonin administration through a mechanism involving ROS inhibition and AMPK activation. To our knowledge, this is the first investigation to demonstrate that the relationship between AMPK-modified mitochondrial injury and ER stress is sufficient to mediate H_2_O_2_-induced oxidative stress injury and that melatonin is an effective agent for promoting BMSC survival.

MSCs, which are important candidates for cell-based strategies, play an important role in promoting tissue repair and regeneration in multisystem diseases because they possess immunosuppressive properties and the capacity for unlimited self-amplification and terminal differentiation [37–39]. In terms of cell characteristics, MSCs isolated from the bone marrow, adipose tissue, or umbilical cord are spindle-shaped, undergo cell growth, and express the surface molecules CD73, CD105, CD90, and CD29 (> 90%) but not CD45, CD34, CD14, or CD79 [40]. However, increasing evidence suggests that the survival of donated stem cells is poor and that stem cell engraftment in the damaged organ fails after delivery, which unavoidably results from a complicated environment involving risk factors that may lead to MSC death, including oxidative stress, and ultimately hinders the clinical application of cytotherapy [41–43]. Therefore, a complete exploration of the underlying mechanisms and drugs with antioxidative effects that enhance MSC migration and survival in the injury-associated microenvironment is crucial for improving both MSC repair capacity and therapeutic applications of MSCs.

Mitochondria, which are known as the principal sources of ROS, are the main organelles involved in metabolism and respiration in eukaryotic cells and are inseparably linked to oxidative stress after disruption of mitochondrial homeostasis [32]. Maintaining the normal function of mitochondria is essential for ensuring the efficient scavenging of ROS and protecting cells from mitochondrion-mediated cell death [44]. Mitochondrial dysfunction, loss of the inner MMP, and increased mitochondrial membrane permeability represent cardinal biochemical markers of apoptosis [45]. Apoptosis induction through a mitochondria-dependent pathway typically involves many regulatory molecules, such as Bcl2, Bax, Cyto-C, and Caspases [46]. In our study, after treatment with H_2_O_2_ (a nonradical derivative of O_2_), ROS production in the cytoplasm and superoxides in mitochondria were increased, as evidenced by DCFH-DA and MitoSOX™ Red staining, respectively; this observation indicated that excessive oxidative stress occurred in the cells; combined with reduced cellular activity and an increase in the number of TUNEL^+^ cells, these results indicated that the mitochondrial apoptotic pathway was activated in BMSCs. Moreover, intracellular oxidative stress depolarizes the MMP, further promoting the recruitment of a proapoptotic protein (Bax) to the mitochondrial membrane, increasing the mitochondrial membrane permeability and thus facilitating the release of Cyto-C [47]. Conversely, the antiapoptotic protein Bcl2 protects mitochondria by inhibiting Bax activation, thus ensuring outer mitochondrial membrane integrity and inhibiting the release of Cyto-C. However, once released, Cyto-C cooperatively activates Caspase-3 and other factors to exacerbate apoptotic cell death [46, 48]; this phenomenon was confirmed by our western blot results.

The ER is an interconnected and continuous network formed by the nuclear envelope and is essentially associated with other organelles in all eukaryotic cells [49]. Structurally, the ER interacts with mitochondria at contact sites called mitochondria-associated membranes (MAMs), which facilitate interorganelle communication [50]. Functionally, the ER is critical for protein synthesis and transport to the extracellular space, mitochondrial function, the maintenance of Ca^2+^ homeostasis, and apoptosis [51]. Thus, the ER interior mimics the relatively oxidized extracellular environment, which causes the ER to be sensitive to perturbations inside or outside the cell, including the oxidative stress response [52]. In this sense, alterations in the oxidation state of the ER lumen can lead to an accumulation of misfolded polypeptides, causing further ER stress [53]. The oxidative folding machinery is then hyperactivated to refold improperly folded proteins; this process is accompanied by the production of ROS and ultimately disrupts ER function for an indefinite period [53]. Therefore, ROS that are mainly generated in mitochondria can perturb ER function; in contrast, under stress conditions, the ER assists with ROS production in mitochondria, leading to a vicious cycle that produces a general state of spiraling oxidative stress. It has been demonstrated that during ER stress, ROS upregulate the levels of protein chaperones (such as Bip and CRT), which play a vital role in the PERK pathway, the signaling pathway that is most closely tied to oxidative stress [52, 54]. Our results demonstrated that H_2_O_2_ increased PERK phosphorylation and the expression of Bip, CRT, p-eIF2a, and DDIT3, which make up the PERK pathway and contribute to signal transmission to regulate cell fate. Moreover, after oxidative stress injury, ER stress in BMSCs is associated with mitochondrial dysfunction and cell death, which may explain the mechanisms underlying ER-mitochondria-induced apoptosis.

ROS produced as part of such a response to perturbations, which act primarily to restore homeostasis, can be considered a part of cellular stress signaling pathways that can mediate AMPK activation by decreasing ATP production [52, 55]. In our study, H_2_O_2_ injury induced AMPK dephosphorylation, possibly because oxidative stress impaired energy metabolism to induce the apoptosis of BMSCs [15]. Additionally, AMPK is an evolutionarily conserved energy sensor that has diverse cytoprotective effects [14, 15]. A number of compounds, including melatonin, have been found to activate and thus biologically regulate AMPK [56].

Melatonin is a hormone derived from tryptophan metabolism. It is mainly synthesized and secreted by the pineal gland and has also been verified to be present at high levels in the bone marrow [19]. Our experiments showed that melatonin promoted the biological behaviors of BMSCs and their ability to maintain self-renewal and differentiation capacities after long-term passage [41], demonstrating that melatonin is a beneficial treatment for cell transplantation. Importantly, our previous findings indicated that melatonin contributes to the amelioration of high-flow shear stress-induced BMSC injury by activating melatonin receptors and AMPK/ACC signaling [4]; however, the specific regulatory mechanisms and effector pathways have not been fully revealed. Considering that melatonin and its metabolites are known to scavenge a variety of free radicals and are able to act both on every cell and within every subcellular compartment [57, 58], we focused on the regulatory effect of melatonin on mitochondria and the ER during H_2_O_2_-induced BMSC injury. Our data showed that melatonin prevented cell death induced by H_2_O_2_ damage, decreased the apoptotic index, and improved cell proliferation. In addition, pretreatment with melatonin relieved oxidative stress and restored mitochondrial function, as evidenced by a decrease in ROS production, an increase in the MMP, inhibition of Cyto-C translocation, and upregulation of MnSOD (an antioxidant enzyme essential for cell survival through the catalysis of the spontaneous dismutation of superoxide radicals [59]). Moreover, melatonin activated AMPK and reduced the expression of proteins related to ER stress, such as p-PERK, Bip, p-eIF2α, and DDIT3. As a result, melatonin exerted an antiapoptotic effect by upregulating Bax and downregulating Bcl2 and Caspase-3 activation.

To explore the relationship between ROS and apoptosis in the protective effect of melatonin against H_2_O_2_ injury, NAC (1 μM) was used in our study. After exposure to NAC, the cytoprotective effects of melatonin on BMSC survival and intracellular ROS were enhanced. Moreover, Apaf-1 plays an essential role in the mitochondrial apoptotic pathway, which is stimulated by Cyto-C to activate Caspase-3 [60]. Melatonin could inhibit the expression of *Apaf-1* at the gene and protein levels after H_2_O_2_ insult, and NAC enhanced the above effects as well as the regulation of other apoptotic proteins (Bcl2, Bax, and Caspase-3). These results indicate that the protective effects of melatonin on cell apoptosis occurred in a mitochondria-dependent manner due to the inhibition of ROS production.

It is worth pointing out that after cotreatment with AICAR, the most commonly used AMPK activator, the protective effects of melatonin on cell proliferation, apoptosis inhibition, and mitochondrial homeostasis were significantly strengthened. In addition, ER stress was further attenuated. Conversely, disrupting the ER with TG in advance abrogated the combined effects of AICAR and melatonin. To further evaluate the relationship between AMPK and ER stress in the process of melatonin-mediated antiapoptotic effects during H_2_O_2_-induced injury, the negative regulatory reagents CpC and 4-PBA (AMPK siRNA and DDIT3 siRNA) were used to downregulate their respective targets. As expected, CpC (AMPK siRNA) markedly blocked the regulatory effects of melatonin on the functions of AMPK, ER, and mitochondria during oxidative stress-induced apoptosis in BMSCs, while 4-PBA (DDIT3 siNA) subsequently and partially restored these effects. This is the first study to comprehensively report the multiple impacts of melatonin on AMPK, the ER, and mitochondria.

## Conclusions

**Fig. 9:**
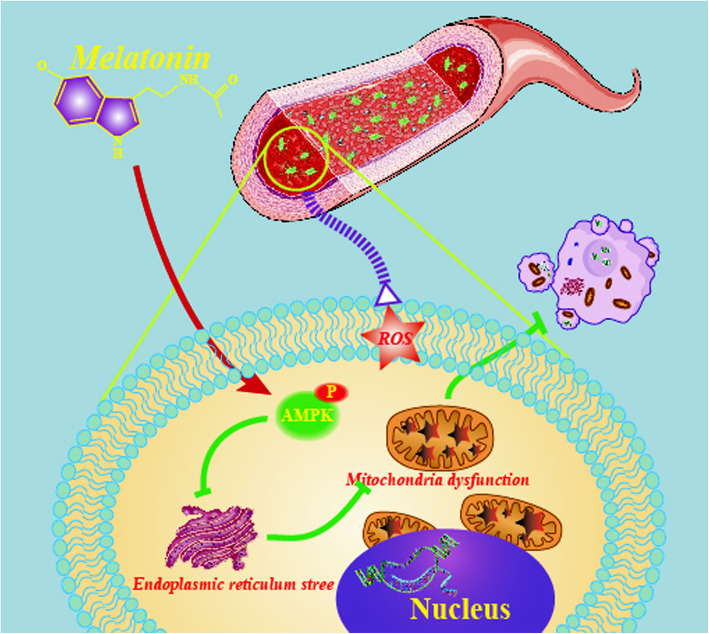
Schematic diagram depicting the likely cellular events through which melatonin recovers mitochondrial homeostasis via the AMPK/ER stress pathway under H_2_O_2_ exposure, resulting in BMSC survival. ER stress-induced mitochondrial dysfunction plays an important role in BMSC oxidative stress injury. Melatonin treatment reduces BMSC damage and improves cell functions after H_2_O_2_ insult. Such cytoprotective effects seem to be largely due to the attenuation of ROS overproduction and the maintenance of mitochondrial homeostasis, which involves AMPK/ER stress signaling pathways, subsequently leading to the inhibition of cell apoptosis

Here, we demonstrate the functional link between AMPK and ER stress in BMSCs and suggest that the activation of AMPK or inhibition of ER stress is a potential target for maintaining mitochondrial homeostasis and promoting cell survival. Our work revealed that melatonin is a good candidate for such effects; it restores AMPK enzyme activity and subsequently suppresses the ER stress-associated mitochondrial oxidative response, thereby inhibiting BMSC apoptosis (Fig. 9). We conclude that melatonin is a physiologically and pharmacologically relevant molecule with remarkable potential for cell-based transplantation.

## Supplementary information

**Additional file 1: Supplementary Figure 1:** Inhibition of cellular oxidative stress on melatonin-mediated cellular protection against H_2_O_2_ damage. BMSCs were pretreated with NAC (1 mM) in the absence or presence of melatonin (100 μM) for 6 h and then exposed to 400 μM H2O2 for another 24 h. The (A) cell viability and (B) ROS production were demonstrated. (C-E) The TUNEL assay and C-Casp-3 immunofluorescence staining were conducted. The percentage of TUNEL^+^ cells and C-Casp-3 fluorescence intensity were indicated to determine the apoptotic degree. Nuclei are labeled with DAPI. (F) *Afpa-1* mRNA expression was analyzed by RT-qPCR. (G) Western blots were used to measure and quantify the expressions of Apaf-1, Bcl2, Bax, and C-Casp-3. Cells treated with PBS were served as a control group. Values are expressed as the mean ± SD (*n* =3 independent experiments). ^*^p < 0.05 compared with the different groups. one-way ANOVA followed by Student’s t-test was used to analyze significant differences. Scale bar = 50 μm. C-Casp-3 (C-C-3, C-C), cleaved Caspase-3; Mel, melatonin; NAC, acetylcysteine; OD, optical density; ROS, reactive oxygen species.

**Additional file 2: Supplementary Figure 2:** Effect of pharmacologic agonists and inhibitors on cell viability in BMSCs. BMSCs were seeded in 96-well plates for 12 h. (A) The experimental protocols were shown about the cotreatment melatonin with AICAR (a), TG (b), CpC (c), 4-PBA (d), AMPKsi or DDIT3si (e) followed by H_2_O_2_ (400 μM) for another 24 h. (B) Different concentrations of AICAR and TG were engaged to incubate for 24 h. The OD values were then analyzed by CCK-8. (C) Western blots were used to measure and quantify the expressions of p-AMPK or p-PERK after treated with AICAR or TG in BMSCs. (D) Different concentrations of CpC and 4-PBA were engaged to incubate for 24 h. The OD values were then analyzed by CCK-8. (E) Western blots were used to measure and quantify the expressions of p-AMPK or p-PERK after treated with CpC or 4-PBA in BMSCs. (F) BMSCs were seeded in six-well plates and transfected with AMPK siRNA (or DDIT3 siRNA). Western blots were used to measure and quantify the expressions of AMPK or DDIT3. (G) Then the cells were incubated with melatonin for 6 h followed by H_2_O_2_ for another 24 h. The OD values were analyzed by CCK-8. Cells treated with PBS were served as a control group. Values are expressed as the mean ± SD (n =3 independent experiments). ^*^p < 0.05 compared with the different groups. one-way ANOVA followed by Student’s t-test was used to analyze significant differences. AICAR, acadesine; AMPKsi, AMPK siRNA; DDIT3si, DDIT3 siRNA; Ctrlsi, control siRNA; CpC, compound C dihydrochloride; Mel, melatonin; OD, optical density; TG, thapsigargin; 4-PBA, 4-phenylbutyric acid.

**Additional file 3: Supplementary Figure 3:** Regulatory effects of activated AMPK or ER stress on melatonin-mediated homeostasis about ROS and mitochondrial function. BMSCs were treated as indicated agents and time to pre-activate AMPK and ER stress. The fluorescent photograph on (A) intracellular ROS, (B) mitochondrial superoxide, and (C) mitochondrial membrane potential were demonstrated (n =3 independent experiments). Scale bar = 50 μm. AICAR, acadesine; Mel, melatonin; TG, thapsigargin.

**Additional file 4: Supplementary Figure 4:** Regulatory effects of inactivated AMPK or ER stress on melatonin-mediated homeostasis about ROS and mitochondrial function. BMSCs were treated as indicated agents and time to pre-inhibit AMPK and ER stress. The fluorescent photograph on (A) intracellular ROS, (B) mitochondrial superoxide, and (C) mitochondrial membrane potential were demonstrated (n =3 independent experiments). Scale bar = 50 μm. CpC, compound C dihydrochloride; Mel, melatonin; 4-PBA, 4-phenylbutyric acid.

## Data Availability

The datasets used and/or analyzed during the current study are available from the corresponding author on reasonable request.

## Abbreviations

4-PBA: 4-Phenylbutyric acid
ACC: Acetyl-Co A carboxylase
AICAR: Acadesine
AMP: Adenosine monophosphate
AMPK: AMP-activated protein kinase
ATF6: Activating transcription factor 6
ATP: Adenosine triphosphate
BCA: Bicinchoninic acid
BM: Basal medium
BMSCs: Bone marrow mesenchymal stem cells
BSA: Bovine serum albumin
C-Casp-3 (C-C-3, C-C): Cleaved Caspase-3
CCK-8: Cell counting kit-8
COX IV: Cytochrome C oxidase IV
CpC: Compound C dihydrochloride
CRT: Calreticulin
Cyto-C: Cytochrome C
DAPI: 4′,6-Diamidino-2-phenylindole
DDIT3: DNA damage-inducible transcript 3
EDTA: Ethylenediaminetetraacetic acid
FBS: Fetal bovine serum
H_2_O_2_: Hydrogen peroxide
HBSS: Hanks balanced salt solution
IRE1: Inositol-requiring protein 1
JC-1: 5,5′,6,6′-Tetraethyl-benzimidazolylcarbocyanine iodide
LDH: Lactate dehydrogenase
MAMs: Mitochondria-associated membranes
Mel: Melatonin
MMP: Mitochondrial membrane potential
NAC: *N*-acetyl-l-cysteine
OD: Optical density
P4: Passage 4
PERK: RNA-dependent protein kinase (PKR)-like ER kinase
p-PERK: Phospho-PERK
PI: Propidium iodide
RIPA: Radioimmunoprecipitation assay
RNS: Reactive nitrogen species
ROS: Reactive oxygen species
RT-qPCR: Real-time quantitative PCR
SD: Standard deviation
siRNA: Small interfering RNA
UPR: Unfolded protein response
TUNEL: Terminal deoxynucleotidyl transferase dUTP nick end labeling
