# Supplementary figures and images for: Melatonin suppresses ER stress-dependent proapoptotic effects via AMPK in bone mesenchymal stem cells during mitochondrial oxidative damage
Source: Stem Cell Res Ther. 2020 Oct 15;11:442. doi: 10.1186/s13287-020-01948-5 (PMC7560057; doi:10.1186/s13287-020-01948-5)

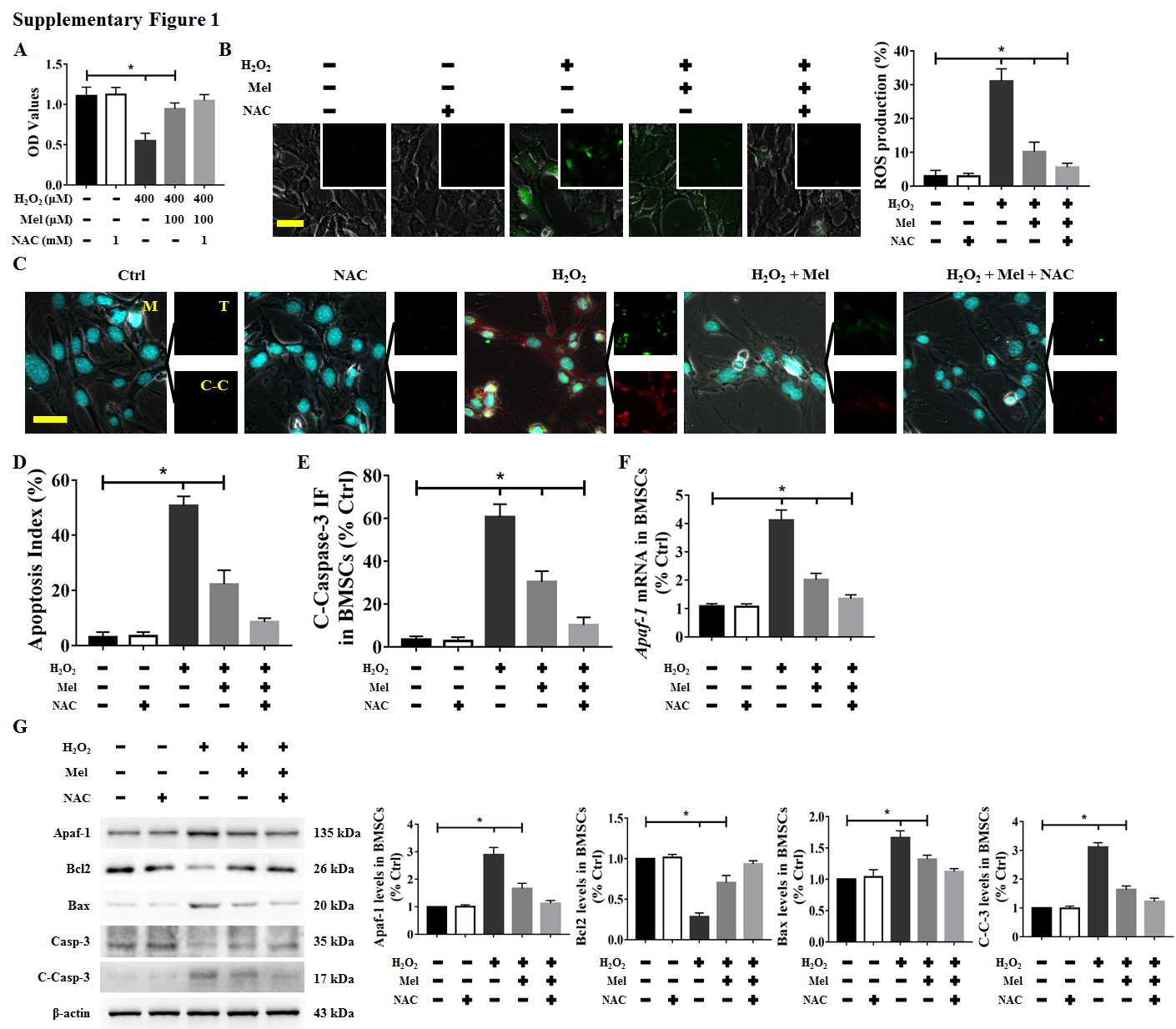

Supplement: Supplementary file 1 — Additional file 1: Supplementary Figure 1: Inhibition of cellular oxidative stress on melatonin-mediated cellular protection against H2O2 damage. BMSCs were pretreated with NAC (1 mM) in the absence or presence of melatonin (100 μM) for 6 h and then exposed to 400 μM H2O2 for another 24 h. The (A) cell viability and (B) ROS production were demonstrated. (C-E) The TUNEL assay and C-Casp-3 immunofluorescence staining were conducted. The percentage of TUNEL+ cells and C-Casp-3 fluorescence intensity were indicated to determine the apoptotic degree. Nuclei are labeled with DAPI. (F) Afpa-1 mRNA expression was analyzed by RT-qPCR. (G) Western blots were used to measure and quantify the expressions of Apaf-1, Bcl2, Bax, and C-Casp-3. Cells treated with PBS were served as a control group. Values are expressed as the mean ± SD (n =3 independent experiments). *p < 0.05 compared with the different groups. one-way ANOVA followed by Student’s t-test was used to analyze significant differences. Scale bar = 50 μm. C-Casp-3 (C-C-3, C-C), cleaved Caspase-3; Mel, melatonin; NAC, acetylcysteine; OD, optical density; ROS, reactive oxygen species. [file 13287_2020_1948_MOESM1_ESM.jpg]

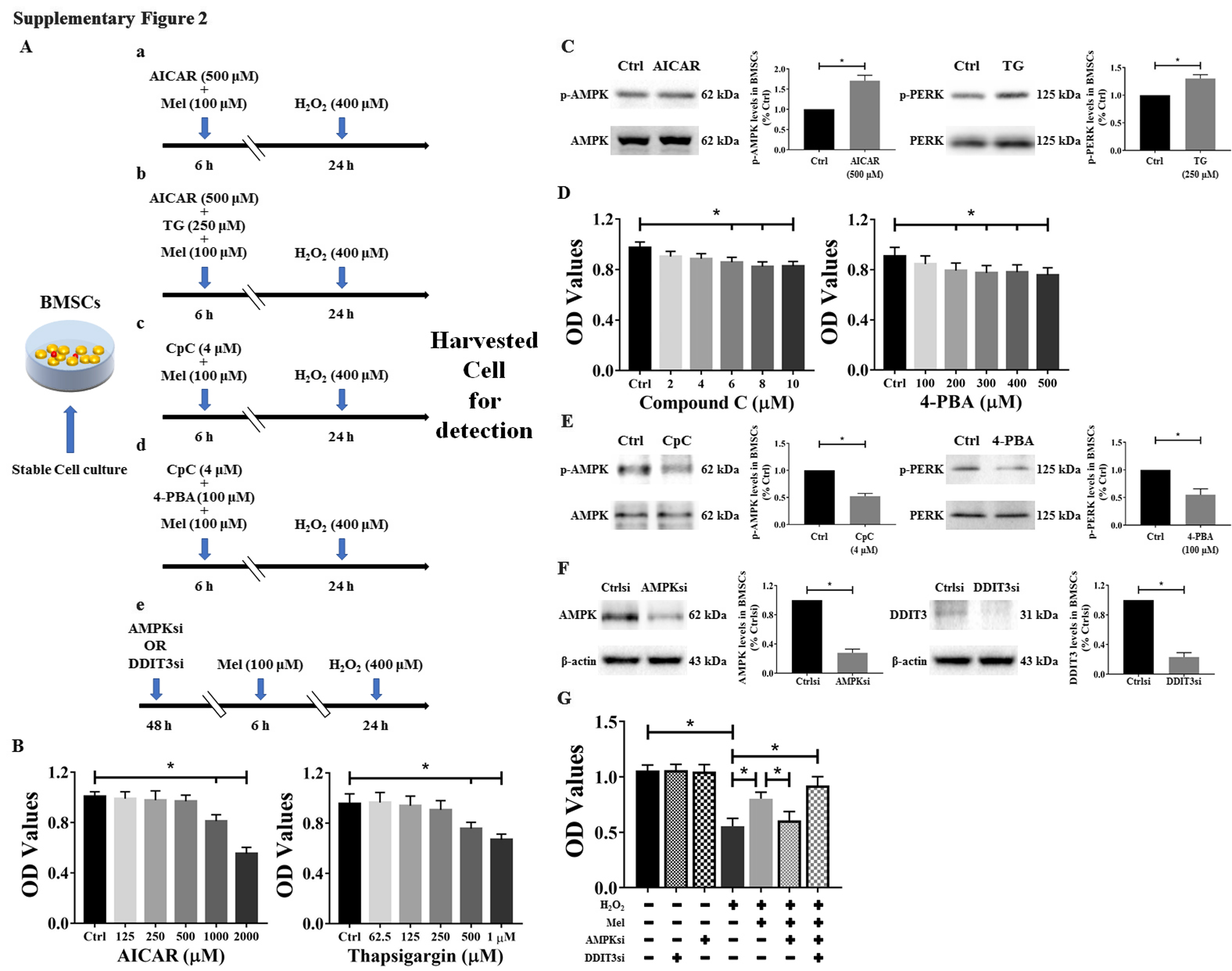

Supplement: Supplementary file 2 — Additional file 2: Supplementary Figure 2: Effect of pharmacologic agonists and inhibitors on cell viability in BMSCs. BMSCs were seeded in 96-well plates for 12 h. (A) The experimental protocols were shown about the cotreatment melatonin with AICAR (a), TG (b), CpC (c), 4-PBA (d), AMPKsi or DDIT3si (e) followed by H2O2 (400 μM) for another 24 h. (B) Different concentrations of AICAR and TG were engaged to incubate for 24 h. The OD values were then analyzed by CCK-8. (C) Western blots were used to measure and quantify the expressions of p-AMPK or p-PERK after treated with AICAR or TG in BMSCs. (D) Different concentrations of CpC and 4-PBA were engaged to incubate for 24 h. The OD values were then analyzed by CCK-8. (E) Western blots were used to measure and quantify the expressions of p-AMPK or p-PERK after treated with CpC or 4-PBA in BMSCs. (F) BMSCs were seeded in six-well plates and transfected with AMPK siRNA (or DDIT3 siRNA). Western blots were used to measure and quantify the expressions of AMPK or DDIT3. (G) Then the cells were incubated with melatonin for 6 h followed by H2O2 for another 24 h. The OD values were analyzed by CCK-8. Cells treated with PBS were served as a control group. Values are expressed as the mean ± SD (n =3 independent experiments). *p < 0.05 compared with the different groups. one-way ANOVA followed by Student’s t-test was used to analyze significant differences. AICAR, acadesine; AMPKsi, AMPK siRNA; DDIT3si, DDIT3 siRNA; Ctrlsi, control siRNA; CpC, compound C dihydrochloride; Mel, melatonin; OD, optical density; TG, thapsigargin; 4-PBA, 4-phenylbutyric acid. [file 13287_2020_1948_MOESM2_ESM.tif]

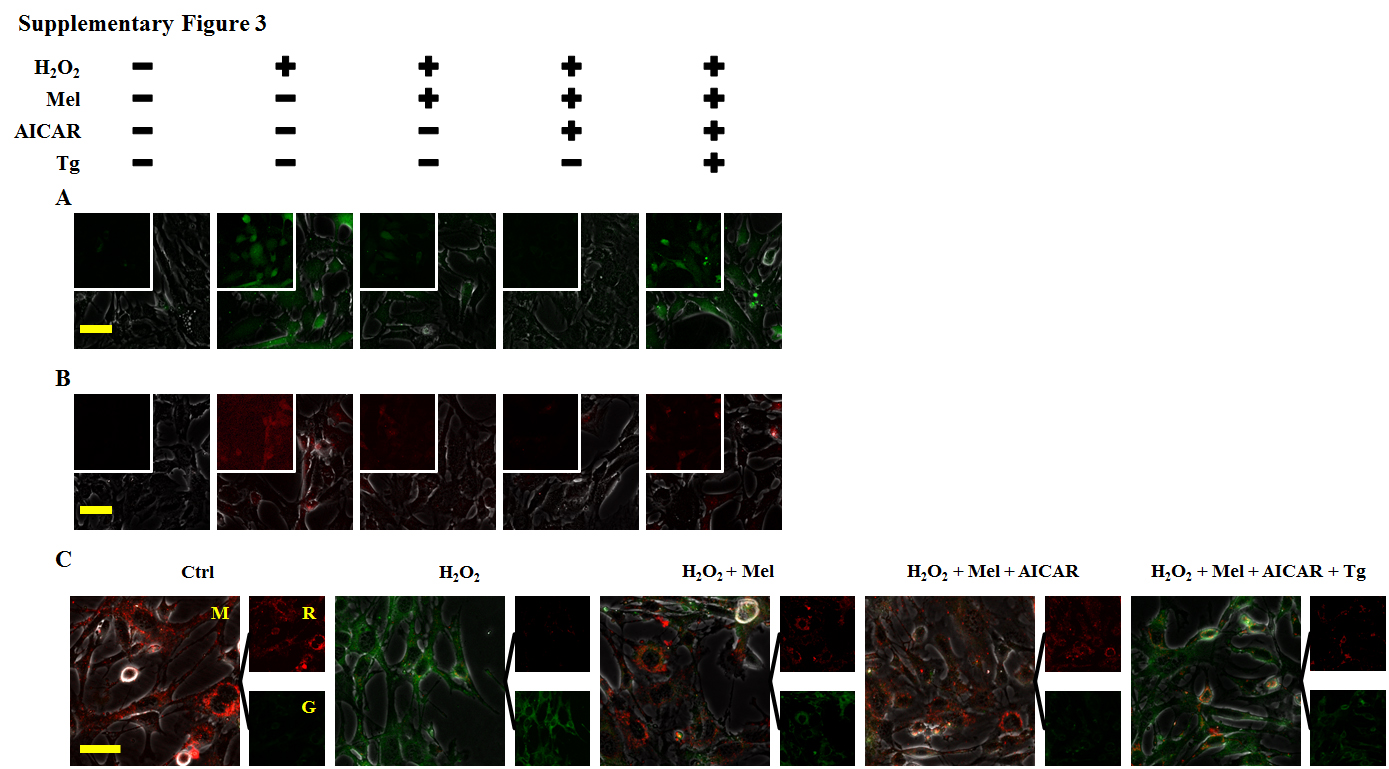

Supplement: Supplementary file 3 — Additional file 3: Supplementary Figure 3: Regulatory effects of activated AMPK or ER stress on melatonin-mediated homeostasis about ROS and mitochondrial function. BMSCs were treated as indicated agents and time to pre-activate AMPK and ER stress. The fluorescent photograph on (A) intracellular ROS, (B) mitochondrial superoxide, and (C) mitochondrial membrane potential were demonstrated (n =3 independent experiments). Scale bar = 50 μm. AICAR, acadesine; Mel, melatonin; TG, thapsigargin. [file 13287_2020_1948_MOESM3_ESM.jpg]

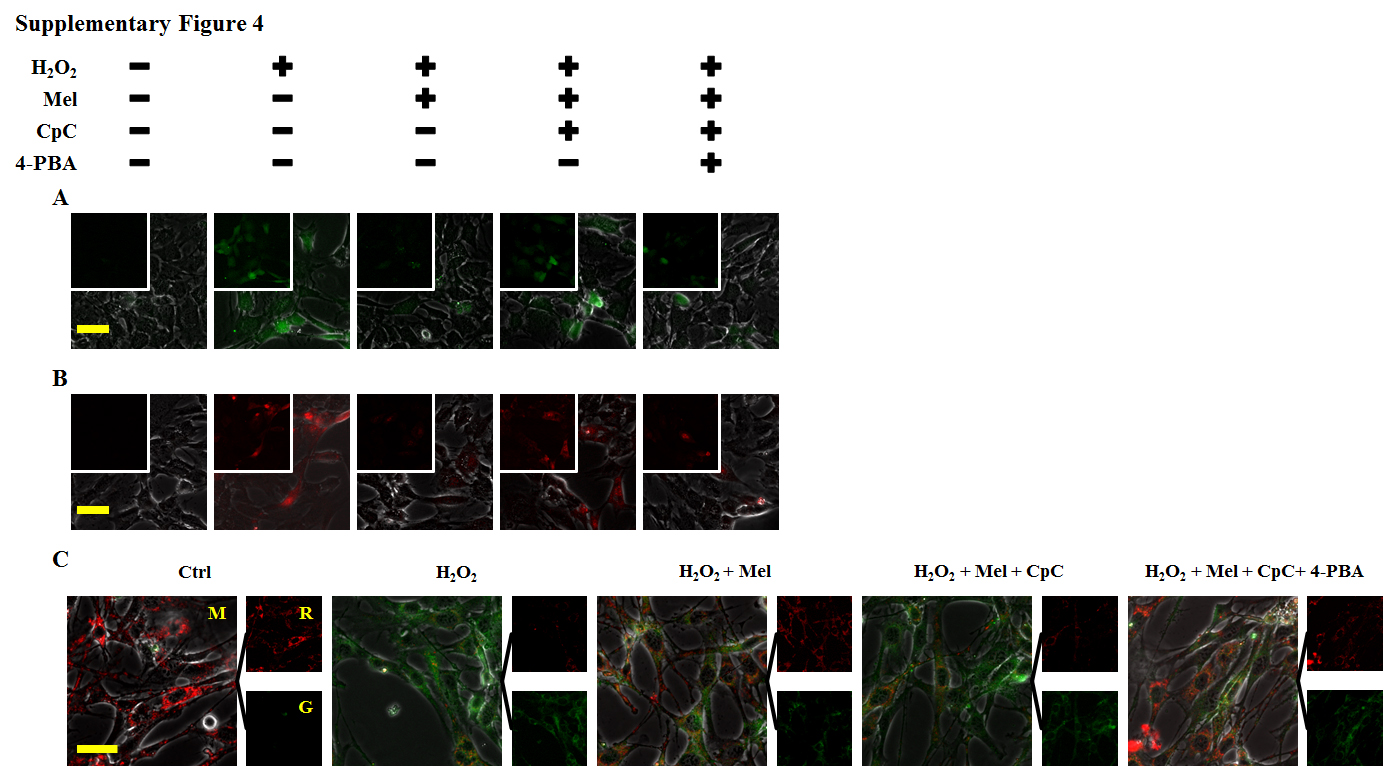

Supplement: Supplementary file 4 — Additional file 4: Supplementary Figure 4: Regulatory effects of inactivated AMPK or ER stress on melatonin-mediated homeostasis about ROS and mitochondrial function. BMSCs were treated as indicated agents and time to pre-inhibit AMPK and ER stress. The fluorescent photograph on (A) intracellular ROS, (B) mitochondrial superoxide, and (C) mitochondrial membrane potential were demonstrated (n =3 independent experiments). Scale bar = 50 μm. CpC, compound C dihydrochloride; Mel, melatonin; 4-PBA, 4-phenylbutyric acid. [file 13287_2020_1948_MOESM4_ESM.jpg]
